# Research Progresses on Nano-Structured Silicon-Based Materials as Anode for Lithium-Ion Batteries

**DOI:** 10.3390/ma18040830

**Published:** 2025-02-14

**Authors:** Xiang Chen, Weidong Cheng, Huanyan Liu, Haiqing Chen, Jiahui Ma, Yihan Zhang, Zhaojun Wu, Chaohui Wang, Yuan You, Xueqing Xing, Zhonghua Wu

**Affiliations:** 1 School of Material Science and Engineering, Qiqihar University, Qiqihar 161006, China; 2 Institute of High Energy Physics, Chinese Academy of Sciences, Beijing 100049, China; 3 College of Chemistry and Chemical Engineering, Qiqihar University, Qiqihar 161006, China; 4 Department of Practice Teaching and Equipment Management, Qiqihar University, Qiqihar 161006, China; 5 University of Chinese Academy of Sciences, Beijing 100049, China

**Keywords:** lithium-ion batteries, silicon-based material, anode, nanostructure designing, composite materials

## Abstract

Silicon-based materials are considered the most promising next-generation anode materials for lithium-ion batteries due to their high theoretical specific capacity, rich reserves, and advantages of low discharge potential. However, the significant volume expansion of silicon during the cycling process leads to the detachment of active substances and the loss of electrical contact between the active substances and the current collector, ultimately resulting in a decline in battery performance. Nanostructured anodes have advantages of high specific surface area, short diffusion path, and the ability to effectively alleviate the volume expansion of electrode material during circulation. Therefore, how to rationally design the nanostructured silicon-based anodes is currently one of the research hotspots. This article first reviews and evaluates the advantages and disadvantages of microstructured and nano-structured silicon anodes in rate performance, discusses cycle stability and volumetric energy density, and discusses and summarizes the lithium storage mechanism of silicon-based materials, with a focus on the influence of some nanostructured silicon anodes and silicon/carbon composites and conductive polymers and silicon/metal composites on the electrochemical properties of materials. Finally, some suggestions and prospects for the future development of silicon-based materials are proposed.

## 1. Introduction

In recent years, the consumption of non-renewable energy has been increasing day by day with the rapid growth of the population. The consumption of traditional fossil fuels still dominates, and their greenhouse gas emissions have a serious impact on the environment [1,2,3,4]. Therefore, exploring renewable energy to achieve energy transition is considered one of the most effective solutions [5,6], and the most critical aspect lies in the storage of renewable energy and resource allocation [7,8]. Energy storage systems mainly include electrochemical energy storage, chemical energy storage, mechanical energy storage, thermal storage, supercapacitor energy storage, and so on. Lithium-ion batteries (LIBs) are one of the most important electrochemical energy storage devices.

LIBs have attracted much attention because of their advantages of high energy density, long cycle life, and low environmental pollution [9,10,11]. They are widely used in various portable electronic devices and have shown great application value in electric and hybrid vehicles. In addition, the performance of electric vehicles is not superior compared with traditional cars, mainly because the energy density of current LIBs has gradually fallen behind the market demand [12], and some high-performance electric vehicles have excessively high research and development costs, which put higher demands on the selection of LIB materials. At present, the cathode performance of LIBs is very close to its theoretical capacity, making it difficult to improve. Therefore, many studies focus on the selection of anode materials [13,14,15,16].

According to its lithium storage mechanism, anode materials can be classified into intercalation type [17], conversion type [18], and alloy type [19]. The representative material of intercalated anodes is carbon-based materials, such as graphite, which has been widely used as anode material for LIBs due to its low cost, good thermal and electrical conductivity, and small volume change during the cycling process [20]. However, it has a lower theoretical capacity (372 mAh/g) because of the limited number of lithium insertion sites [21]. The conversion anode is mainly composed of metal oxides and sulfides, which can cause oxidation-reduction reactions between Li^+^ and electrode material at a certain potential, converting them into metal elements and lithium compounds, and the redox reactions are reversible. The theoretical capacity of most conversion anodes is about 1000 mAh/g, such as Fe_2_O_3_ (1007 mAh/g) [22], Mn_2_O_3_ (1019 mAh/g) [23], Co_3_O_4_ (890 mAh/g) [24], MoO_3_ (1100 mAh/g) [25]. However, its practicality is limited by its high working potential, low Coulomb efficiency, and voltage hysteresis. The alloy anode represents tin-based and silicon-based materials, and its working principle is that lithium ions and the material itself form an alloy compound to store lithium ions. Silicon-based materials have attracted much attention due to their ultra-high theoretical specific capacity (4200 mAh/g), abundant reserves (26.4%), and low discharge potential (~370 mV) [26]. Nevertheless, the excessive volume expansion of silicon-based materials in the cycle processes affects their direct use as negative electrode materials, so it is necessary to mitigate the structural changes during lithiation through reasonable structural design.

Excellent nanostructures have significant advantages in high specific surface area, short diffusion channels, fracture toughness, and fatigue resistance [27]. Therefore, designing silicon-based anode materials of LIBs with excellent nanostructure morphology can effectively alleviate the structural changes during lithium deintercalation, reduce the stress and strain generated, and improve their cycling stability [28,29,30].

**Figure 1:**
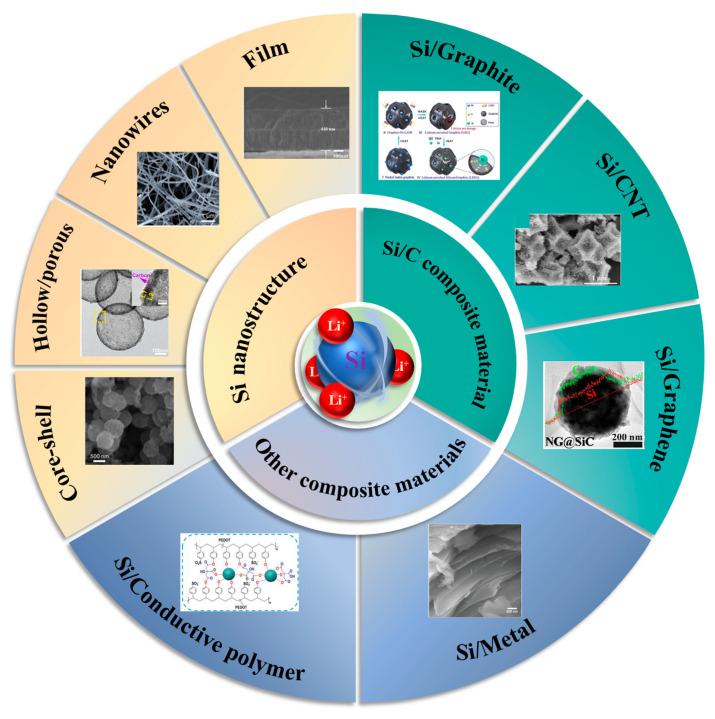
Modification strategy of silicon-based anode materials for lithium-ion batteries.

In recent years, there have been many excellent papers on the review of silicon-based anode materials [31,32,33], mainly focusing on synthesis methods, silicon–carbon composite materials, nanostructured silicon anode design, silicon anode mechanism failure study, and pre-lithium. However, there are some omissions in the reported literature, including the classification of nanostructured silicon anodes is not detailed enough, and the analysis of the lithium storage mechanism and structure of silicon anodes provides few modification methods. At the same time, novel preparation methods and good data need to be recorded, and the advantages and disadvantages of nanostructures and microstructures need to be understood for readers to analyze more intuitively. In this paper, the latest research progress of nanostructured silicon-based materials as anodes of LIB is reviewed. Firstly, the advantages and disadvantages of nanostructured and microstructural anodes are briefly discussed. The differences in electrochemical properties such as rate performance, cycle stability, and volumetric energy density were explained in detail in terms of particle size, diffusion path, specific surface area, stress-strain, and first loop coulomb efficiency. Then, some modification studies on the nanostructure of silicon-based materials and some composites synthesized with different carbon sources are highlighted. At the same time, the positive effects of the addition of conductive polymers and silicon/metal composite materials on the modification of silicon anode cannot be ignored (Figure 1). Among them, the rational design of nanostructures, excellent mechanical properties of carbon materials, the binder synthesized by conductive polymers, the modification of the stability of SEI film, and the high electrical conductivity of metals all contribute to the stable improvement of the electrochemical performance of silicon anodes. Finally, some suggestions and prospects for the future development of silicon-based materials are proposed.

## 2. Nanostructure/Microstructure and Lithium Mechanism of Si Anode

### 2.1. Nanostructure/Microstructure

An important reason for affecting the energy density of LIBs comes from the particle size of the material, and whether it should be designed as a nanostructure or a microstructure has been debated by researchers. For alloy-type anode materials, the time required for lithium ions to diffuse into the active material particles and undergo an alloying reaction is faster in nanoscale materials than in micrometer-scale materials [34]. In terms of speed performance, nanoscale particles have a larger electrode contact area and smaller diffusion paths compared to micrometer-sized particles at high current densities, ensuring an increase in electrode power density. Due to the influence of particle size, the stress and strain generated during the lithium extraction and insertion of micron-sized particles are greater than those of nano-sized particles, and they cannot be uniformly lithiated, resulting in poor cyclic stability. Some studies have pointed out that when the diameter of silicon particles is above 150 nm [35], the stress accumulation generated during the cycle can destroy the particle structure, resulting in cracking and falling off. In addition, lithium ions and electrolytes can be continuously consumed to generate solid electrolyte interphase (SEI). The generated SEI will also continue to crack and then continue to be generated in the cracked part, which will result in uneven thickness of SEI while reducing capacity, hindering the diffusion of lithium ions. When it is below this critical size, silicon particles can resist the accumulation of stress without the need to counteract stress and strain through particle rupture, indicating that nanoparticles have higher mechanical stability. In addition, nanoparticles have a higher specific surface area than micron particles, which can provide more active sites, store more lithium ions, and have a higher specific capacity [36].

It cannot be ignored that nanostructured anodes still have many problems. The high specific surface area of nanoparticles will consume more lithium ions and electrolytes than that of microparticles during the formation of SEI in the first cycle, thereby reducing the first coulombic efficiency. The smaller the nanoparticle size, the higher their surface energy activity, which inevitably leads to agglomeration and promotes the occurrence of side reactions. In terms of volumetric energy density, nanoparticles have a lower volumetric energy density compared to microparticles, which makes it difficult to meet industry standards. However, designing reasonable nanostructures is still an effective strategy to solve many problems at the silicon level. At present, a large amount of structural design work has been carried out to study and improve the electrochemical performance of silicon-based anodes in response to the problems of volume expansion and poor conductivity of silicon-based materials.

### 2.2. Lithium Mechanism of Si Anode

Because of its stable chemical properties, silicon is difficult to react with other substances at room temperature. As a negative electrode of LIBs, it belongs to the alloy type and reacts with lithium ions to form alloy compounds (Li_x_Si) in different proportions during the process of lithium extraction and insertion. Four intermediate equilibrium phases will gradually form at high temperatures, namely Li_12_Si_7_, Li_7_S_i3_, Li_13_Si_4_, and Li_22_Si_5_ [37], which exhibit multiple voltage platforms on the charge and discharge curves. The Li_22_Si_5_ phase is a lithium-rich phase formed by 4.4 Li atoms and one silicon atom, with the maximum theoretical specific capacity (4200 mAh/g). The final phase formed at room temperature is Li_15_Si_4_, which is a metastable phase with a theoretical specific capacity of 3589 mAh/g [38]. In the initial discharge process, amorphous Li_x_Si is formed by an alloying reaction between Li^+^ and Si atoms, and the intensity of the crystalline Si peak decreases with the continuous lithiation process. When the electrode potential drops below 60 mV vs. Li/Li^+^, amorphous Li_x_Si gradually transforms into crystalline Li_15_Si_4_ [39]. In the subsequent process of de-lithium, Li_15_Si_4_ will gradually transform into amorphous Li_x_Si with the continuous removal of Li^+^, and the entire process is reversible. However, the formed silicon is amorphous rather than the initial crystalline state, and it remains in an amorphous state throughout the entire cycle. The process of lithium insertion and extraction is as follows (c is crystalline, a is amorphous):

**Lithium insertion**

Si (c) + xLi^+^ + xe^−^→Li_x_Si (a)

Li_x_Si (a) + xLi^+^ + xe^−^→Li_15_Si_4_ (c)

**De-lithiation**

Li_15_Si_4_ (c)→xLi^+^ + xe^−^ + Si (a)

## 3. Nanostructured Silicon

### 3.1. Core–Shell Structure

In a narrow sense, core–shell structure refers to a structure formed by a shell composed of one or more layers of materials enclosing the core inside. Applying it to silicon anodes can effectively alleviate the problem of volume expansion during cycling, maintain structural integrity, avoid direct contact of electrolytes with active substances, and reduce the occurrence of side reactions [40]. The synergistic effect between the outer shell and the inner nano silicon particle core can bring about new or enhanced chemical and physical properties, depending on the choice of outer shell material [41]. Li et al. [42] prepared TiO_2_-coated nano-silicon particles by calcination and introduced silver nanowires (Ag NWs) to improve the conductivity of the anode material. The TiO_2_ shell provides mechanical support, suppresses the volume expansion of silicon during the cycling, and avoids direct contact between the silicon nuclei and the electrolyte, reducing the repeated generation of SEI film. Ag NWs have excellent electrical conductivity, which can improve the electronic conductivity of the entire composite material, thereby improving the electrode stability. The SiNPs@TiO_2_/AgNWs material exhibits excellent electrochemical performance with an initial specific discharge capacity of 3524 mAh/g at a current density of 400 mA/g. To improve the stability of the silicon anode, He et al. [43] coated the surface of silicon particles with a layer of N and S co-doped carbon shell through in situ polymerization and pyrolysis of pyrrole, in which heteroatoms N and S are derived from pyrrole and initiator persulfate. The introduction of heteroatoms could provide abundant defect sites, expand the interlayer spacing of carbon lattice, and enhance the conductivity of the material. The outer carbon layer improves the electrical conductivity while ensuring the structural stability of the core silicon particles. After cycling 550 times at a current density of 0.3 A/g, the capacity of the Si@NSC electrode remains at 1720 mAh/g with a retention rate of 90.2%. After increasing the current density to 0.5 A/g and cycling 550 times, it still has a reversible capacity of 743 mAh/g, demonstrating excellent electrochemical performance. When the current density is increased to 0.5 A/g for 550 cycles, it still has a reversible capacity of 743 mAh/g, demonstrating excellent electrochemical performance.

**Figure 2:**
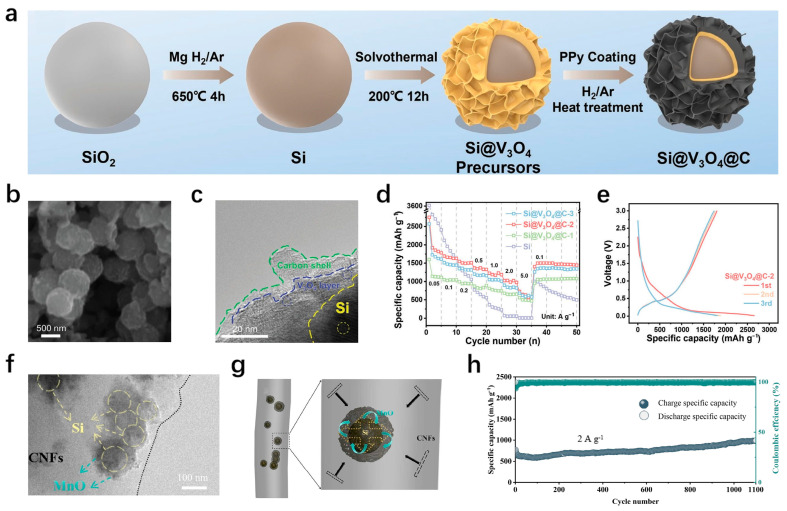
(**a**) Schematic illustration of the synthesis process for the Si@V_3_O_4_@C composites. (**b**) TEM images of Si@V_3_O_4_@C-2. (**c**) SEM images of Si@V_3_O_4_@C-2 (**d**) Rate performance of Si@V_3_O_4_@C-1/2/3 and Si. (**e**) Galvanostatic discharge–charge profiles of Si@V_3_O_4_@C-2 [44]. (**f**,**g**) TEM images of Si@MnO@CNFs-2. (**h**) Cycling performance of Si@MnO@CNFs-2 at 2 A g^−1^ [46].

The core–shell structure with a single shell sometimes cannot cope with the stress accumulation generated during long-term lithium extraction and insertion processes, and may still experience capacity degradation due to fracture and active material detachment, ultimately leading to electrode failure. The coordination between multiple shells can effectively improve this situation. Wang et al. [44] reasonably designed A double-layer coating structure composed of V_3_O_4_ and C layers was reasonably designed through solvothermal reaction. Figure 2a shows the schematic illustration of the synthesis process. Si particles were obtained by Mg thermal reduction of SiO_2_, and then Si@V_3_O_4_@C precursor was prepared by solvent-thermal reaction, finally annealed to form Si@V_3_O_4_@C. Figure 2b,c shows the topography of the sample. It can be seen that the V_3_O_4_ layer and the carbon layer successively envelop the spherical SiO_2_. After 700 cycles at a current density of 0.5 A/g, the prepared electrode has a capacity of 1061.1 mAh/g, a capacity retention rate of 70%, and an average Coulomb efficiency of 99.3%. Figure 2d,e shows the rate performance and the charge–discharge curves of the sample. In the full-battery test with a cathode of LiFePO_4_, its capacity retention rate is 78.7% after 130 cycles at 0.5 C, and this demonstrates the excellent potential of the prepared electrodes. Thanks to its reasonable structural design, V_3_O_4_, as the intermediate layer, not only ensures the structural stability of Si particles during the cycle but also accelerates the diffusion rate of lithium ions with its multi-tunnel structure. The outer carbon layer provides high electrical conductivity, allowing for efficient electron transfer. Moreover, the double shell can buffer the mechanical strain caused by long-term cycling and ensure the structural integrity of Si particles.

The other sample is a Si@SnO_2_@C composite material with a double shell structure prepared by hydrothermal method [45], which has an initial discharge capacity of 2777 mAh/g at a 0.1 A/g current density. After 300 cycles at a 0.5 A/g current density, the capacity is 1104.2 mAh/g with a capacity retention rate of 50.2%. The middle layer of SnO_2_ will gradually generate Sn during the circulation process, which can play the role of connecting the external carbon layer and the internal silicon, accelerate the transmission of ions and electrons, and improve the circulation stability of the electrode. It can be seen that the material selection of the outer shell is crucial, which can play a good role in solving the problems of silicon volume expansion and poor electrical conductivity. Among them, the selected intermediate layer material should coordinate the relationship between the outermost layer and the inner core, accelerate the free shuttle of ions and electrons, and play the role of “high-speed channel”. To improve the cyclic stability of silicon anodes at high current densities, Zhang et al. [46] prepared a double-shell coated silicon-based material Si@MnO@CNFs by liquid phase method and electrospinning technology (Figure 2f,g). Its capacity is 1593.7 mAh/g after 1000 cycles at a current density of 1 A/g and remains at 994.45 mAh/g after 1100 cycles even at a high current density of 2 A/g (Figure 2h). This is due to the excellent performance brought by its unique structure. The CNTs in the outer layer can shorten the transport path of lithium ions, and MnO, as the intermediate layer, has high theoretical capacity and electrochemical kinetics. In addition, it is also very important to design the proportion of each component in the silicon-based materials. Silicon is a contributor to the high capacity of the electrode, and how to balance the subsequent structural instability caused by high silicon content and the limited capacity provided by less silicon content needs to be studied and discussed.

### 3.2. Hollow/Porous Structure

#### 3.2.1. Hollow Structure

**Figure 3:**
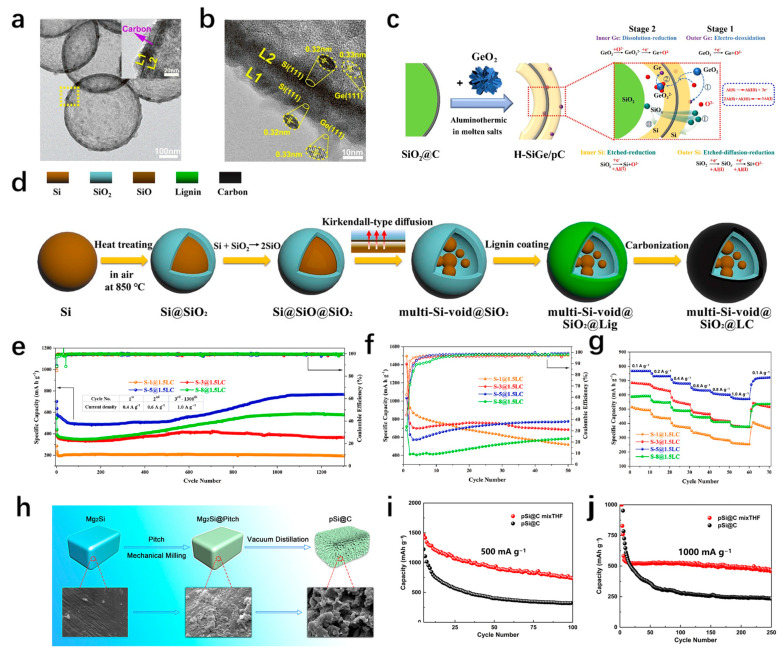
(**a**) TEM (the inset is the enlarged region of yellow rectangle) and (**b**) HRTEM images of SiGe-10%. (**c**) Schematic illustration of the H-SiGe/pC formation by simultaneous diffusion and reduction mechanisms in molten salts [50]. (**d**) Scheme of the formation mechanism of the multi-Si-void@SiO_2_@LC structure. (**e**) Cycle performances of S-1@1.5LC, S-3@1.5LC, S-5@1.5LC and S-8@1.5LC electrodes at 0.1 A/g. The samples were activated at 0.1 A/g for 50 cycles and then the (**f**) rate performances and (**g**) cycle performances at 1.0 A/g were tested [52]. (**h**) Schematic representation of preparation of pSi@C. The cycling capability of pSi@C at (**i**) 500 mA g^−1^ and (**j**) 1000 mA g^−1^ [53].

The hollow structure is often used to improve the silicon anodes because of its unique internal cavity structure. Its larger specific surface area increases lithium storage sites, while the tensile stress of hollow silicon spheres is much smaller than that of solid silicon spheres. Its large internal void can effectively cope with the volume changes, and the thinner shell can shorten the ion diffusion path and improve the electrochemical performance [47,48,49]. Gao et al. [50] synthesized the hollow silicon sphere heterostructure H-SiGe/pC by a low-temperature aluminothermic reduction process. The hollow silicon balls are formed due to the inconsistency of internal and external diffusion rates caused by the regulation of the middle porous carbon layer (Figure 3a,b). Ge nanodots are evenly embedded in the silicon shell and distributed on both sides of the carbon layer, showing a sandwich structure. Figure 3c shows its structural morphology and diffusion reduction diagram. The H-SiGe/pC anode has excellent electrochemical performance, with an initial specific capacity of 2922.2 mAh/g at 0.1 A/g current density, a capacity of 1215.6 mAh/g after 200 cycles at 4 A/g, and a 1255.4 mAh/g capacity even after 10 cycles at a high current density of 8 A/g. SiO_2_ can be reduced to Si by a magnesiothermic reduction reaction, and then some by-products are removed by HCl etching to form hollow silicon spheres [51]. The hollow silicon spheres are surrounded by amorphous carbon formed by magnesium reduction, which can provide excellent electrical conductivity. After 200 cycles of 0.5 A/g current density, the capacity retention rate is as high as 65.7%. In addition, different from the hollow silicon sphere, the hollow structure formed by reserving a certain gap between the inner core and the outer shell can also effectively alleviate structural deformation.

Based on the Kirkendall effect, the multi-Si-void@SiO_2_@LC material of multi-core and internal void structure was synthesized by a simple heat treatment and acid precipitation process (Figure 3d) [52]. Under the current density of 1 A/g, the electrode capacity remained at 759 mAh/g after 1300 cycles (Figure 3e), indicating that it has good cycle stability. They believe that stable performance should come from the coordination between the components of the composite. The design of this multi-core structure enables the inner core to have a higher contact area with the SiO_2_ intermediate layer. The hydrogen bond interaction between the lignin and SiO_2_ in the outer layer strengthens the mechanical properties of the material, and the reserved space inside prevents excessive stress concentration, which effectively responds to the volume changes. The irreversible Li_4_SiO_4_ and Li_2_O products produced in the cycle processes have high mechanical strength and excellent ionic electronic conductivity. In short, the rational structural design from inside to outside is the key to promoting high cyclic stability. To determine the optimal ratio of reserved space and silicon content, several test samples were designed through different calcination times to find the best proportion (Figure 3f,g). It can be seen that the advantages of the hollow structure come from the ability to reduce the increased stress endured during the cycle process, and can accelerate the ion diffusion and shorten the ion diffusion path, so as to improve the anode cycle stability.

#### 3.2.2. Porous Structure

The porous silicon-based anode can reduce the stress generated during the cycle processes due to its unique advantages of three-dimensional structure (Figure 3h), and multiple channels of the porous structure can accelerate the diffusion rate of lithium ions and improve the cycle stability of the electrode (Figure 3i,j) [53,54]. Its applications in silicon-based materials can be roughly divided into nanoporous silicon and micron-porous silicon with nano properties. Studies have shown that the critical fracture diameter of porous silicon nanoparticles is 1.52 µm [55], which is much larger than that of crystalline or amorphous silicon particles. This suggests that porous silicon has a better capacity to accommodate volume changes.

At present, the common methods for synthesizing porous silicon are the etching method and the template method. Yan et al. etched nano silicon particles with urea, and then coated the surface with a layer of amorphous carbon by CVD method, and obtained N-PoSi@C material with evenly distributed voids [56]. The preparation process is simple, green, and low-cost. After 200 cycles at a current density of 0.4 A/g, the discharge capacity of the electrode is 1645.6 mAh/g, and the capacity retention rate is 81.6%. In the tests of high current densities, the electrochemical cyclic stabilities of the material are also very excellent. The discharge capacity is 861.5 mAh/g after 800 cycles at the current density of 4 A/g, and remains 596.3 mAh/g after 1000 cycles at the high current density of 10 A/g. The structures of composites prepared by different processes are often different. Wada et al. [57] prepared 3DNP-Si by the dealloying method, that is, porous silicon was formed by removing metal elements from silicon metal alloy. Bi-metal melt was used to remove Mg elements from the Si-Mg alloy, and HNO_3_ was used to etch Bi in the gap to form a composite. The 3DNP-Si sample is composed of many connected nano-silicon particles surrounded by many interconnected nano-pores, which can accelerate the transport of lithium ions and effectively adapt to the volume change in the (de)intercalation of lithium. At the same time, the large specific surface area can store more lithium ions. The electrochemical performance test shows that the discharge capacity of the electrode prepared by this sample is 3550 mAh/g at 1/2 C (current density 1800 mA/g). In addition, porous silicon can be prepared using SiO_2_ as a template [58]. Using low-cost rice husks as silicon sources, porous nano-silicon material coated with graphitized carbon was prepared by low-temperature magnesium thermal reduction and acid etching process. The synthesized material inherits the natural form of rice husks and contains a large number of pores, which is conducive to the transmission of lithium ions and improves the reaction kinetics. The external graphitized carbon generated by CO_2_ reduction improves the conductivity and Coulomb efficiency of the material, which makes it have good cycling performance.

### 3.3. Silicon Nanowires and Nanotubes

**Figure 4:**
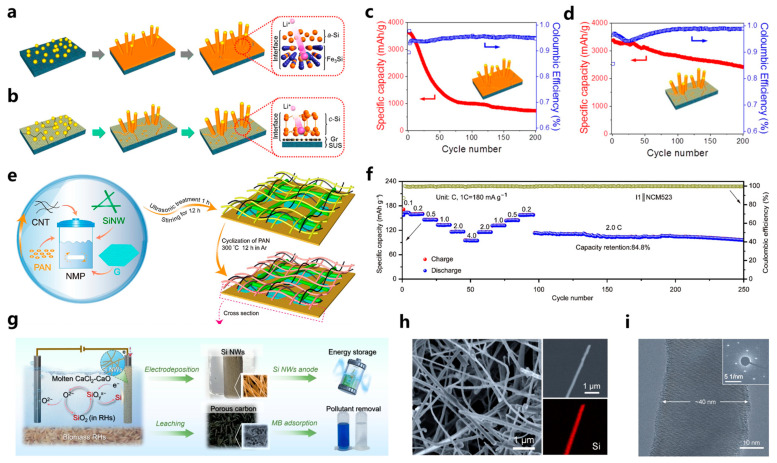
Schematic diagrams illustrating different scenarios for Si NW anodes on metal current collectors in LiBs, with and without graphene interfacial layers. (**a**) CVD-grown SiNWs on a current collector lead to the formation of parasitic Si layers, in which cracks and delamination occur during LiBs cycling. Interfacial reaction and Li^+^ ion diffusion occur at the interface between the Si layer and the current collector. (**b**) CVD-grown Si NWs on graphene without parasitic Si layers. No cracks occur after cycling. Interfacial reactions and Li^+^ ion diffusion are blocked by graphene. (**c**) Si NW anode and (**d**) SiNW-Gr anode during 200 cycles at the charging and discharging rate of 0.1 C [64]. (**e**) Schematic illustration depicting the fabrication process and intricate structure of the integrated Si electrode. (**f**) Rate performance and cycle performance of I1||NCM523 [66]. (**g**) Schematic illustration of the sustainable conversion of RHs into Si NWs and porous carbon materials with applications in energy and environmental sustainability. (**h**) SEM images of Si NWs. (**i**) TEM images and SAED pattern of the as-synthesized Si NWs [67].

Silicon nanowires have great advantages in cycle stability and rate performance of LIBs. Their one-dimensional conductive path provides fast charge transfer. During the alloying processes of anode material, radial and axial expansion can be carried out to alleviate the stress and deformation [59,60]. Silicon nanowires can be grown directly on the current collector [61] without conductive agents and binders, which increases the silicon content in the electrode and exhibits good mechanical strength. To avoid the fracture of silicon nanowires caused by the stress generated by alloying during the cycle processes, Nguyen et al. [62] prepared a highly interconnected silicon nanowire on a stainless steel film using gold catalyst by plasma-enhanced chemical vapor deposition technology. Due to the particularity of the gas–liquid–solid (VLS) growth mechanism, the Au-Si liquid alloy at the tip of the metal wires tends to bend and grow towards the high-temperature region (substrate region). The generated silicon nanowires can be highly entangled to form an interconnected network by adjusting the substrate temperature. This compact structure improves the cyclic stability of the electrode material, and the capacity retention rates are 100% and 90% after 40 and 70 cycles at C/2 and 2 C ratios, respectively. To deal with the instability of the electrode interface in the process of circulation, silicon nanowires can be grown by using a copper silicide nanofoam collector (3D Cu_x_Si_y_ NF) [63]. The monolayer interconnected copper silicide network grown on copper foil has a higher area load, and the inherent characteristics of nanowires are conducive to high-density homogeneous deposition of small-size catalyst seeds, which can effectively promote the growth of high-density silicon nanowires. After the electrochemical performance tests, the metal compound sample has effective fracture resistance and can well cope with the adverse reactions during the cycle. In addition, Xia et al. [64] generated graphene sheets on copper foil by CVD, and then grew silicon nanowires on the sheets. It can be seen from Figure 4a,b, that the prepared SiNW-GR anode can effectively inhibit the formation of silicon islands or silicon film in the growth stage of nanowires due to the introduction of graphene, which greatly reduces the capacity loss caused by the volume change of silicon islands during the cycle process, and improves the cycle stability. Compared with Si NW electrode, the cycle stability of the SiNW-GR anode is improved by more than 170%, the capacity of 200 cycles at 0.1 C is maintained at 2400 mAh/g, and the Coulomb efficiency is 99% (Figure 4c,d). At the same time, the graphene interface improves the electrical contact and alleviates the continuous cracking and recombination of the SEI film caused by stress accumulation. The impedance change in the SINW-GR anode is smaller than that of the Si NW electrode (more than 140% after 5 cycles), and the impedance change in the SiNW-GR anode is less than 70% after 5 cycles.

It is also an effective strategy to use the synergy between different materials to dope or compound with Si NWs. Stokes et al. [65] synthesized Si-Ge nanowires with axial heterostructure as anode material. Ge has an excellent lithium ion diffusion rate and electrical conductivity. It can effectively alleviate the problem of poor conductivity and volume expansion of silicon nanowires during the cycle. The heterostructure can enable Si and Ge to play their respective characteristics. After 400 cycles at C/5 and 10 C, the discharge capacity is 1180 mAh/g and 613 mAh/g, respectively, showing good rate performance. Li et al. [66] prepared a stable three-dimensional silicon anode G/SiNW@CNT/cPAN using a cyclic process of polyacrylonitrile (PAN). As shown in Figure 4e, PAN forms elastic and electrically conductive pyridine ring encapsulated silicon at lower temperatures, and then combines with carbon nanotubes (CNT) and graphite sheets with high electrical conductivity and good mechanical properties, further enhancing the cyclic stability of the electrode and the rapid transport of ionic and electrons. At the same time, the total impedance of the anode is reduced and the electrochemical performance is improved due to the presence of PAN without binder. The capacity of the anode maintains at 650 mAh/g after 1000 cycles at 3 A/g, and its capacity is 886 mAh/g at 8 A/g rate test. The electrode expansion rate is only 13.3% after 20 cycles at 2 A/g current density, which is attributed to the good structural stability and the excellent synergy of the components in the G/SiNW@CNT/cPAN composite. In the full battery test, the capacity retention rate of the synthesized material is 84.8% after 160 cycles at a rate of 2 C. It shows outstanding rate performance and valuable market prospects (Figure 4f). In addition to direct growth, silicon nanowires can also be obtained by etching technology. Pang et al. [67] converted rice husk (RH) into silicon nanowire and porous carbon by the molten salt leach–electrodeposition method (Figure 4g), used molten salt to leach SiO_2_ in RH and converted it into soluble silicate ions, and finally extracted the silicon nanowire by electro reduction and the silicon deposition of silicate. The morphology and characteristics of silicon nanowires extracted in the whole process are adjustable (Figure 4h,i). The silicon nanowires as anodes have a reversible specific capacity of 2410 mAh/g at a current density of 0.1 A/g, showing good cycling performance.

Compared with the one-dimensional structure of silicon nanowires, the hollow structure of silicon nanotubes can better adapt to the volume changes during the cycles and improve the cycle stability [68]. The internal voids of silicon nanotubes can provide additional free interface, and will not produce additional external surfaces to accelerate the formation of SEI films, which ensures the stability of SEI film and reduces capacity loss [69]. The preparation process of silicon nanotubes is cumbersome. The common preparation methods are the template method or the application of a catalyst to synthesize silicon nanotubes [70,71]. Alexander et al. [72] prepared porous silicon nanotubes on a stainless steel substrate using the ZnO nanowire template method. Due to the advantages of a large specific surface area and a three-dimensional porous structure, the electrode has a discharge capacity of 3095 mAh/g at a current of C/20, and the capacity remained at 1670 mAh/g after 30 cycles. To improve the contact between silicon nanotubes and the substrate interface, Wang et al. [73] chose carbon cloth substrate (CC) and used carbon to encapsulate silicon nanotubes to address the volume expansion issue and enhance the material conductivity. They pointed out that depositing a coating with a low volume expansion rate on the surface of silicon nanotubes can release stress during the cycling inward rather than outward, which is very beneficial for protecting the stability of the SEI film. After 100 cycles at a rate of 0.5 C, the discharge capacity of the hybrid electrode was maintained at 3654 mAh/g.

**Figure 5:**
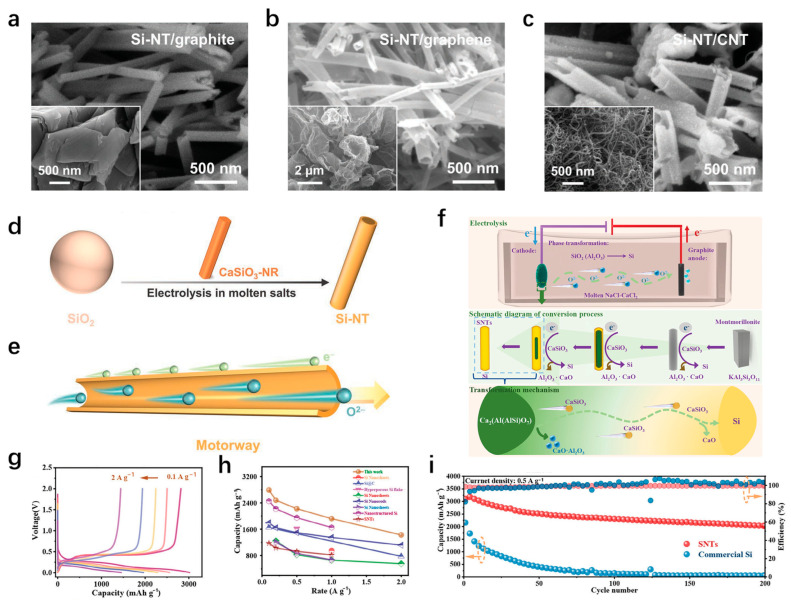
SEM images of (**a**) Si-NT/graphite, (**b**) Si-NT/graphene, and (**c**) Si-NT/CNT obtained from electrolysis of the composites at 2.0 V for 4 h at 800 °C (inset: SEM images of graphite, graphene and CNT parts). (**d**) the electrochemical reduction of SiO_2_ to Si-NT and (**e**) the motorway for fast transport of e^−^ and O^2−^ along/within NTs [74]. (**f**) Illustrations of the formation mechanisms of SNTs. (**g**) dischage–charge curves of SNTs electrode. (**h**) Comparison of rate performance of Si-based electrode. (**i**) Long cycling of SNTs electrode [75].

In addition, the preparation of silicon nanotubes by the fusion electrolysis method is favored because of its low cost, high efficiency, and no need for templates and catalysts. Jing et al. [74] produced silicon nanotubes by the electroreduction of silica particles by melting CaCl_2_-NaCl at 800 °C. CaSiO_3_ nanorods were converted from silica electrolyzed in molten salt and were the precursor of silicon nanotubes. With the subsequent extraction of oxygen ions and calcium ions in the electrolysis process, silicon nanotubes are finally formed. Considering the poor conductivity of silicon dioxide, the researchers added a certain proportion of carbon-based materials (graphite, graphene, and carbon nanotubes, Figure 5a–c) to silicon dioxide to improve the electrolysis efficiency. One-dimensional (1D) CaSiO_3_ nanorods (CaSiO_3_-NR) are intermediates of SiO_2_ molten salt electrolysis, and silicon nanotubes synthesized using CaSiO_3_-NR as a template accelerate the rapid electron transport due to its unique structural advantages (Figure 5d,e). The electrochemical performance of the prepared electrode is enhanced significantly, and the energy consumption is reduced by 30% compared with that of industrial silicon. Wang et al. [75] used montmorillonite as a precursor to prepare silicon nanotubes by melting electrolysis (Figure 5f). The montmorillonite has a uniform layered structure with a spacing of 1–2 nm, which is conducive to the release of oxygen ions in the electrolytic process and improves the electrolytic rate. The synthesized silicon nanotube electrode has excellent electrochemical performance, with a high capacity of 2791 mAh/g at 0.2 A/g, and a discharge capacity of 1427 mAh/g at 2 A/g (Figure 5g). The synthesized electrode also showed better magnification performance compared to the previously reported silicon-based material (Figure 5h). After 200 cycles of 0.5 A/g (Figure 5i), the capacity remained at 2045 mAh/g, which benefits from the good hollow structure of the silicon nanotubes, the volume expansion of the electrode during the cycle is alleviated, and the excellent electrochemical performance is shown.

### 3.4. Silicon Film

The thickness of silicon films is generally between tens of nanometers and several microns, which effectively shortens the diffusion paths of lithium ions and electrons and improves the rate performance [76]. Moreover, it can be used directly as an anode without the binders, which increases the silicon content in the material and reduces the increase in internal resistance caused by the addition of insulating binders [77]. However, when the thickness of silicon film reaches a critical size, the capacity attenuation caused by volume expansion will still occur [78], and its inherent low conductivity will also affect its practical use. Therefore, it is necessary to avoid or reduce the impact of these problems as much as possible through reasonable structural design.

**Figure 6:**
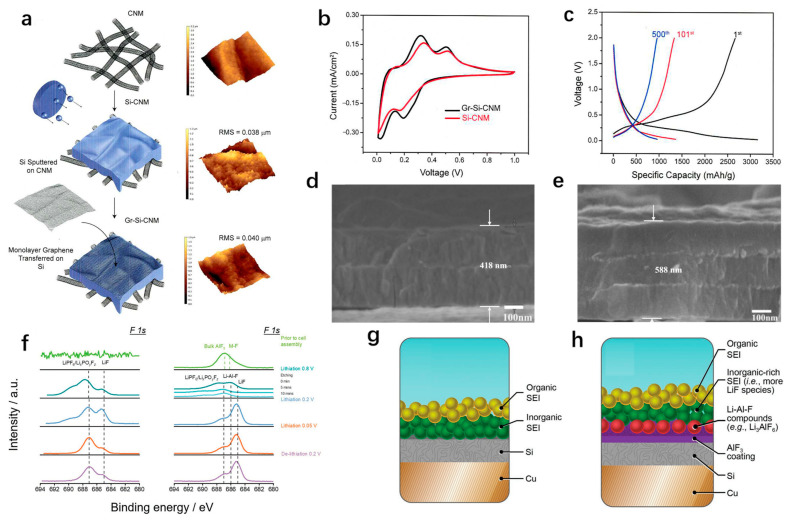
(**a**) Schematic representation of Gr-Si-CNM synthesis. Si was sputtered on a CNM film, and a monolayer graphene sheet was then transferred onto Si-CNM by wet chemistry methods. (**b**) Cyclic Voltammograms (CV) of Si-CNM and Gr-Si-CNM at a scan rate of 0.1 mV/ps. The CV scans shows that silicon is the active anode material in both Si-CNM and Gr-Si-CNM. (**c**) Galvanostatic charge/discharge curves of Gr-Si-CNM plotted after 1, 100, and 500 cycles [82]. Cross-sectional SEM images of (**d**) D_SiAg_3_, and (**e**) T_SiAg_3_ [83]. XPS core spectra of the F 1s: (**f**) uncoated Si, and 20 nm AlF_3_-coated Si thin films. Schematic illustrations of the SEI on (**g**) uncoated Si and (**h**) coated Si electrodes [85].

The main methods for preparing silicon thin films are chemical vapor deposition (CVD) and physical vapor deposition (PVD) [79]. A three-dimensional graphene scaffold was synthesized on nickel foil using microwave plasma enhanced chemical vapor deposition (MPCVD), and then silicon was deposited onto it to synthesize a silicon–graphene hybrid electrode [80]. The electrode has an initial capacity of 1560 mAh/g at a current density of 0.797 A/g. After 500 cycles, the retention rate is 84%. The specific capacities are 1083 and 803 mAh/g after 1200 cycles at 2.39 A/g and 7.17 A/g, respectively. This indicates that the stress generated during the cycle can be released and absorbed by using an elastic substrate [81], which has a significant effect on increasing the thickness of the silicon film and improving the bulk energy density of the electrode. Suresh et al. used carbon nanotubes as a substrate and graphene-coated silicon film to stabilize the SEI film generated during the cycles [82]. Figure 6a shows the schematic representation of Gr-Si-CNM synthesis. Firstly, floating catalyst chemical vapor deposition is applied to synthesize a substrate composed of double-walled carbon nanotubes. Carbon nanotube substrates, due to their good flexibility compared to copper foil, can reduce stress accumulation during cycling and prevent the cracking of active substances. Subsequently, the silicon film was synthesized on the carbon nanotube substrate by radio frequency sputtering. Finally, a thin layer of graphene was deposited on the surface. Compared with silicon film deposited on copper foil, the electrochemical performance of the composite anode was greatly improved. The capacity is maintained at 650 mAh/g after 1000 cycles at a current density of 1.8 A/g, and the Coulomb efficiency is 99.67%. When compared with the CV data of the sample without the addition of Graphene (Si-CNM), it can be seen that the CV results of the Gr-Si-CNM sample are consistent with the original, indicating that the addition of graphene only increases the interfacial charge transfer dynamics of the material, but does not contribute to the charge accumulation during the cycle (Figure 6b), which is also confirmed in the voltage-capacity curve (Figure 6c). The increased current density also shows that the addition of graphene can improve the electrochemical reaction kinetics of the electrode. In response to the problem of poor conductivity of silicon thin films, in addition to adding carbon materials to improve conductivity, metal particles can also be added to improve it. Chen et al. [83] improved the rate performance of SiAg_3_ composites by adding highly conductive silver nanoparticles between two/three layers of amorphous silicon films (Figure 6d,e). The electrochemical performance of the silicon film is closely related to its thickness. The SEI film formed during the cycling processes of thinner silicon anode (20 nm thickness) shows a stable and parabolic growth. As the thickness of the film increases, the continuous cracking of the silicon film leads to a linear growth trend in the SEI film. The thicker the film, the more serious the deterioration of its performance, but the addition of silver nanoparticles can effectively alleviate the adverse effects of the upper and lower silicon layer interface. In addition, silver nanoparticles can well stabilize the SEI film formed on the silicon surface during the cycling processes, enhance the conductivity of composite materials, promote Li^+^/e^−^ transport, and thus improve the cyclic stability of silicon anode [84]. The capacity of the material reached 1250 mAh/g at 10 C, and the capacity retention rate was 46%. To demonstrate the improvement effect of silver nanoparticles, the thickness of the single-layer silicon film was set above the average crack spacing (200 nm). The results show that the higher the density of silver nanoparticles (with a surface coverage of 65%), the better the electrode rate performance, which is attributed to the charge-induced electric field condensed around the silver nanoparticles accelerating the diffusion of lithium ions. It can effectively promote the lithium reaction. At the same time, the authors believe that the volume energy density of the anode can be increased by expanding from the nanoscale to the submicron structure without a significant decrease in rate and cycle stability.

Forming a stable SEI film during lithiation is a favorable strategy to reduce capacity decay, but the understanding of SEI film is not yet complete. Adhitama et al. [85] conducted a detailed study on the effect of depositing a layer of AlF_3_ on the interface of a silicon thin film anode. The XPS results before cell assembly and after cycling to different (de-)lithiation states in the first cycle are shown. The cycled samples are held at 0.8, 0.2, 0.05 V (discharge), and 0.2 V (charge) (Figure 6f). The experiment results show that the intermediate phase Li-Al-F formed by the AlF_3_ during the cycle processes facilitates the diffusion of Li^+^, thereby decreasing the charge transfer resistance. This is because the presence of the Li-Al-F phase reduces the energy barrier for Li^+^ embedding and enhances the charge transfer dynamics. At the same time, the transformation of the AlF_3_ phase to the Li-Al-F phase generates a large amount of LiF that can inhibit the continuous decomposition of SEI film, which improves the cycle stability of silicon film (Figure 6g,h), and provides a direction for subsequent research on surface coating modification of silicon films. At the same time, the comparison of electrochemical properties of some nanostructured silicon anodes mentioned above is summarized in Table 1.

## 4. Silicon Carbon Composite Material

The superior electrical conductivity and good mechanical properties of carbon-based composite materials can effectively reduce the adverse effects of silicon anodes due to poor electrical conductivity and volume expansion [86]. At present, there have been a lot of reports on various carbon materials (graphite [87], graphene [88], carbon nanotubes [89], biomass carbon [90], MOFs-derived carbon [91], MXenes, etc. [92]) for compounding with silicon anodes. Different types of carbon materials have some common characteristics, namely excellent mechanical properties and good electrical conductivity, which can be a good target for the volume expansion of silicon materials and low electrical conductivity, improving the cycle stability and rate performance of silicon anodes.

### 4.1. Silicon/Graphite Composites

**Figure 7:**
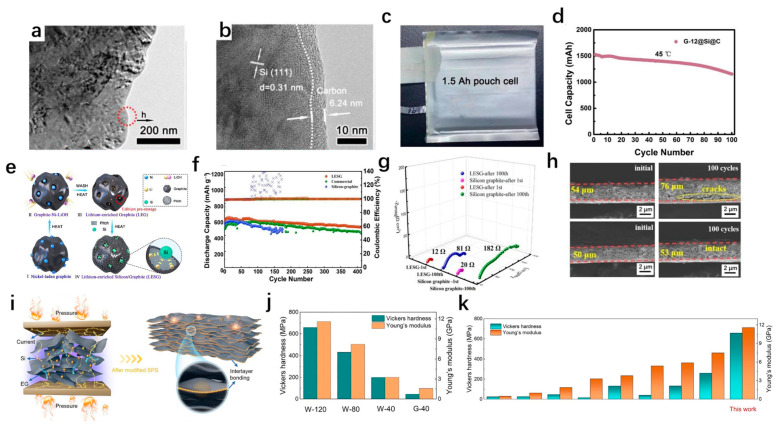
(**a**,**b**) HRTEM images of the G-12@Si@C. (**c**) Photograph of the 1.5 Ah pouch cell. (**d**) Discharging capacity retention of the G-12@Si@C/NCM for 100 cycles at 1 C (discharge) and 0.5 C (charge) at 45 °C [95]. (**e**) Schematic of the lithium pre-storage and the fabrication process of LESG. (**f**) Cycling performances of LESG, commercial silicon-graphite, and silicon-graphite cells. (**g**) Nyquist plots of the LESG and silicon-graphite cell after first cycle and after 100 cycles. (**h**) Cross-sectional SEM images of silicon–graphite electrode and LESG electrode before cycling and after 100 cycles [97]. (**i**) Schematic diagram of Si and EG composite blocks prepared by modified SPS technology. (**j**) Vickers hardness and Young’s modulus results of the as-prepared anode materials from the indentation test. (**k**) Comparison of W-120 with reported Si-based electrodes in Vickers hardness and Young’s modulus [98].

Graphite has been commercialized due to its low cost, high conductivity, small volume changes during cycling, and high Coulomb efficiency. However, its low capacity (372 mAh/g) has limited its commercial value [93]. The reasonable combination of graphite and silicon materials can give full play to the advantages of both, and at the same time, it is necessary to avoid the adverse effects of their respective shortcomings, which puts forward the requirements for reasonable structural design. Li et al. [94] synthesized a low-silicon core–shell structure composite material with graphite as the core and silicon nanoparticles filled in the porous carbon formed by pyrolysis of citric acid and asphalt as the shell by spray drying and pyrolysis. The low-silicon core–shell composite has excellent electrochemical performance the initial discharge capacity is 723.8 mAh/g, and the capacity remained at 592.4 mAh/g after 100 cycles at 0.1 A/g. In general, to avoid the drastic structural changes and cycle performance degradation caused by the excessive silicon content in the composite material, the proportion of doped silicon will not be too much, but this will reduce the energy density of the composite electrode to a certain extent. To increase the silicon content and reduce a series of negative effects caused by high silicon content, Yan et al. [95] prepared flake graphite by the sanding grinding method, and then prepared a silicon/graphite composite with a high silicon content (31.6%) by the CVD method. Finally, carbon coating was carried out to obtain the G@Si@C composite. The silicon deposited by the CVD method is evenly distributed on the flake graphite, which avoids the side reaction caused by aggregation. The large specific surface area of the flake graphite is beneficial for depositing more silicon, improving energy density, and avoiding the formation of larger SEI and silicon nanoparticles. As shown in Figure 7a,b, the external carbon coating avoids direct contact between the electrolyte and the silicon. The G@Si@C electrode has an initial Coulomb efficiency of 86.3% and a capacity retention rate of 94.3% after 100 cycles. The cycle performances are tested at room temperature (25 °C) and high temperature (45 °C) in the 1.5 Ah bag-type full battery test (active substance density of 1.6 g/cm^3^, area capacity of 3.46 mAh/cm^2^) (Figure 7c,d). At room temperature, the capacity is maintained at 96% after 50 cycles at 1 C, and the average Coulomb efficiency is greater than 99.5%. The capacity retention rate is 76% after 100 cycles at 1 C at high temperature, showing excellent rate performance.

In addition to increasing the energy density of the electrode, the low initial Coulomb efficiency of the silicon anode also needs to be paid attention to, and pre-lithiation is a good solution. Prelithiation can compensate for the irreversible loss of lithium ions during the first charge and discharge process, improve the cycle performance, and enable the electrode to work continuously at a lower potential, which is conducive to the continuous utilization of graphite in composite materials and reduces volume expansion [96]. Gao et al. [97] prepared LEG (Li-rich graphite) with rich lithium functional groups on the graphite surface by metal-assisted nano-drilling method and LiOH etchant, and then combined nano-silicon powder, asphalt, and LEG to form LESG composite material through high-temperature sintering. Figure 7e shows the schematic of the lithium pre-storage and the fabrication process. The LESG sample is porous and spherical, while the nano-silicon particles are evenly distributed in the pores. The EDS test finds the aggregation of Li elements in the graphite and nano-silicon regions, which is attributed to the lithium storage capacity of silicon at high temperatures. The stored lithium can be released during the first cycle to make up for the lithium loss, which greatly improves the first Coulomb efficiency of the anode. The first-coulomb efficiency of the LESG electrode is 116%. Meanwhile, this can be seen from Figure 7f that the LESG cell exhibits a stable cycle of more than 400 cycles due to the unique design of the composite structure. The presence of a porous structure and peripheral pyrolytic carbon layer can improve the conductivity of the electrode and form a stable SEI film. The volume of the LESG composite increases by only 6% after 100 cycles (Figure 7g,h), which highlights its excellent cycling performance.

In addition, Lai et al. [98] chose expanded graphite (EG) to composite with nano-silicon. Firstly, the Si EG composite with the ratio of EG to nano-silicon of four to one was prepared by the ball milling method, and then the Si EG powder was used to induce the interlayer bonding of EG in the spark plasma sintering system, due to the double action of high current and pressure, the edge of EG will gradually form a closed structure, and eventually cover silicon ions to obtain the final product (Figure 7i). The modified EG has larger interlayer spacing, higher mechanical properties (high Young’s modulus and Vickers hardness) (Figure 7j,k), and a higher proportion of non-planar sp^2^ and sp^3^ bonding. This structure is not only conducive to the insertion and extraction of Li^+^, but also can effectively reduce the internal mechanical stress and ensure stable SEI film growth. In the electrochemical performance tests, the capacity is stable at 739 mAh/g after 100 cycles at a current density of 0.1 A/g, the capacity retention rate is 94%, and the capacity decay rate is only 0.06% per cycle. The capacity retention rate is as high as 94% after 100 cycles at a current density of 2 A. Even at a high mass load of 4.8 mg/cm^2^, the capacity remains at 632 mAh/g after 100 cycles at 0.1 A/g, and its output capacity is 5 times higher than that of commercial graphite anode at 2 A/g, which reflects its excellent cycle performance and rate performance and has the potential for practical applications.

### 4.2. Silicon/Graphene Composites

Graphene is a two-dimensional structure composed of a layer of sp^2^ hybrid carbon atoms. Due to its high electrical conductivity, high elastic modulus, large specific surface area, and high electron transfer rate, graphene has been widely used in electrochemical fields [99,100,101]. Li et al. [102] used microwave plasma-enhanced chemical vapor deposition technology to encapsulate silicon nanoparticles in nanosheets composed of graphene to accommodate the volume changes of silicon nanoparticles. The graphene nanosheets grow vertically on the substrate, and the silicon nanoparticles are encapsulated inside. The anode does not require a bonding stage and has good rate performance with a capacity of 412 mAh/g at 8 A/g. Its three-dimensional structure is conducive to the rapid transport of lithium ions and electrons and improves cycle stability. Park et al. [103] used the high conductivity and elasticity of graphene to design a hollow structure composed of graphene to replace conventional conductive carbon black to directly combine with silicon. Firstly, graphene oxide is prepared by spray drying method, and then the thermal reduction of the graphene oxide is carried out at high temperatures. The structure expands from the fold structure into a spherical hollow structure with the removal of oxygen. This hollow graphene structure can flatten the free voids around the shell and absorb the stress during the lithiation process. Compared with the original conductive agent, the strong mechanical properties of hollow graphene structures can improve the cycling capacity and mechanical properties of the anode.

**Figure 8:**
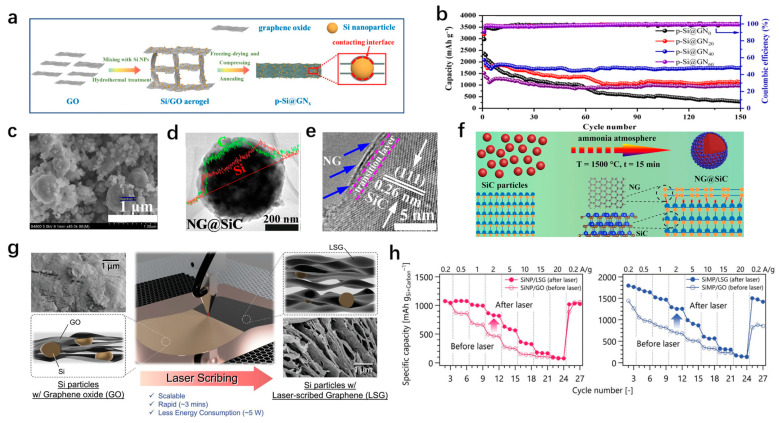
(**a**) Schematic diagram of the preparation procedure of p-Si@GNx. (**b**) Cyclic performances of the p-Si@GNx samples at 300 mA/g [104]. (**c**) SEM, (**d**) TEM (inset: linear elemental scan analysis), and (**e**) HRTEM images of NG@SiC. (**f**) Schematic illustration of synthetic process of NG@SiC [105]. (**g**) Conceptual illustration of the laser-scribing method. (**h**) Specific discharge capacities of SiNP/GO and SiNP/LSG and SiMP/GO and SiMP/LSG at various current densities [106].

To improve the synergy between silicon and graphene composites, reduce the instability of the contact interface caused by size factors, and improve the effective transfer of electrons on the contact interface, Ma et al. [104] synthesized a close-packed composite pSi@GNx by the high-pressure compression and annealing of porous silica/graphene aerogel obtained by hydrothermal and freeze-drying. The surface of the silicon nanoparticles is tightly covered by graphene through pressure compression, which improves its electrical conductivity (Figure 8a). The tightly packed state reduces the direct exposure of silicon nanoparticles in the electrolyte and avoids the overgrowth of SEI film. For graphene, the layer spacing is reduced due to the pressure, and the π-π conjugated system is partially restored, which increases the intermolecular force and can effectively alleviate the propagation of cracks during the cycle. The anode capacity obtained after 150 cycles at a 0.3 A/g current is 1730 mAh/g, and has a capacity retention rate of 82.6% (Figure 8b). The capacity is 706.4 mAh/g at a 3 A/g current density. The above data highlights the excellent rate performance of the pSi@GNx anode. This provides another strategy for improving the interface effect and the anode performance. Similarly, by adjusting the silicon anode interface, Sun et al. [105] prepared the NG@SiC electrode material and achieved the tunable interaction of the atomic interface by the epitaxial growth of N-doped graphene on SiC (Figure 8c–e). High-quality epitaxial graphene layers have higher electrical conductivity. After annealing in ammonia at high temperature, the covalent bond between the interfacial N-doped graphene layer and SiC is passivated and can be used as an electron/ion bridge, which accelerates the interfacial charge migration and enhances the reaction kinetics (Figure 8f). In addition, it was found through first-principles calculations that the formation of the epitaxial N-doped graphene layer reduces the diffusion barrier from 0.84 eV of the original SiC to 0.43 eV, which further confirms that the designed interface is conducive to charge transfer and improves the cyclic stability and rate performance of the NG@SiC anode. The anode maintained a high reversible capacity of 1197.5 mAh/g after 200 cycles at a current density of 0.1 A/g. At a high current density of 10 A/g, there is still a discharge capacity of 447.8 mAh/g after 1000 cycles, and the capacity retention rate was 76.6%. The synthesized NG@SiC electrode has great potential for achieving high energy density and long cycle life in LIBs.

To reduce the complexity of the preparation method and quickly and conveniently composite silicon with graphene, Katsuyama et al. [106] used laser etching technology to etch graphene and compound it with silicon (Figure 8g). The well-formed 3D graphene network structure is conducive to the rapid transmission of ions; compared with the original Si nanoparticles (SiNPs) and Si microparticles (SiMPs), the rate performance and cycle stability of SiNP/GO (before laser scribing) and SiMP/LSG (Si/laser-scribed graphene) were improved (Figure 8h). This method can have a porous 3D graphene network within a few minutes, and the energy consumption is low, which has great research prospects.

### 4.3. Silicon/Carbon Nanotube Composites

Carbon nanotubes (CNTs) can realize point-line-surface contact with silicon materials to ensure the rapid transmission of electrons with a minimum amount of addition, and their one-dimensional tubular structure has a high aspect ratio and can form a long-distance conductive network [107]. As a carbon atom material with an sp^2^ bond, the ultra-high flexibility and strength of CNTs can well absorb the stress generated during the silicon anode cycle and reduce the volume change [108]. Jin et al. [109] prepared Si@N-doped CNT composites by the pyrolysis process, in which carbon nanotubes were formed by the thermolysis of the co-zeolite imidazole skeleton (ZIF-67) as the precursor, and silicon nanoparticles were initially embedded in ZIF-67. The carbon nanotube network has a rich pore structure with a pore size ranging from 3 to 10 nm, which can well restrain the cyclic stress deformation and increase the diffusion rate of lithium ions. The Si@N-doped CNTs electrode can provide a high discharge capacity of 1144 mAh/g at a current density of 1 A/g for 750 cycles, showing high energy density and good cycle stability, which is attributed to the excellent flexibility and conductivity of carbon nanotubes. This advantage of CNTs can be used directly as a current collector, conductive agent, and binder. Conventional copper foil is easy to deform during the cycle, resulting in active substances falling off and capacity attenuation, and the high flexibility of CNTs can reduce the adverse effect caused by metal current collectors. Lee et al. [110] dispersed silicon and BaTiO_3_ (BTO) nanoparticles into multi-walled CNTs by the ball milling process. The CNTs act as the matrix to inhibit the deformation, silicon nanoparticles provide high specific capacity, and BTO nanoparticles generate piezoelectric potential through mechanical stress during the cycle, which increases the carrier mobility and improves the electrochemical properties. The good coordination of the three factors improves the cycle stability of the silicon anode. Fan et al. [111] combined PVDF-derived carbon and commercial CNTs as all-carbon binders with silicon particles to compare their advantages with traditional manufacturing processes (PVDF plus carbon black). The addition of CNTs enhances the mechanical properties and electrical conductivity of the composite. Compared with traditional raw materials, the composite effect of PVDF-derived carbon and CNTs makes the electrode have more bonding active substances and the “binder” bonding ability of the current collector, which strengthens the internal relationship between the anode and current collector to a certain extent, improves the cohesion and structural stability of the composite, and increases the electrical contact.

**Figure 9:**
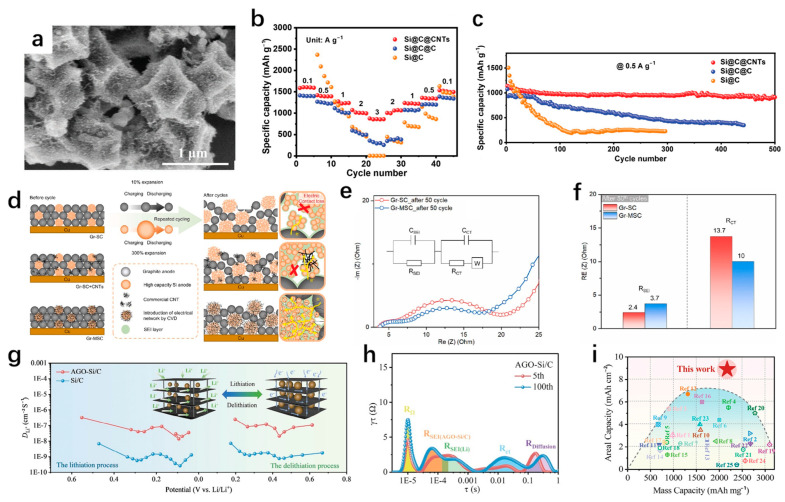
(**a**) SEM images of Si@C@CNTs (**b**) Rate capability of the Si/C composites. (**c**) Cycling stability of the Si@C@CNTs, Si@C@C, and Si@C at 0.5 A g^−1^ [112]. (**d**) Schematic illustration for influence of additional electrical network on blended electrode fabricated with graphite and Si-based material during cycling (**e**) Nyquist plots for Gr-SC and Gr-MSC after 50 cycles. (**f**) RCT and RSEI values measured in Gr-SC and Gr-MSC at 50 cycles [113]. (**g**) The D_Li_+ of both AGO-Si/C and Si/C electrodes in different charged/discharged conditions, inset is the schematic illustration of the Li^+^ and electron pathway in biomimetic AGO−Si/C structure during lithiation and delithiation. (**h**) Corresponding DRT transition result of Li||AGO-Si/C battery based on EIS. (**i**) Comparison of the electrochemical performance between the biomimetic AGO-Si/C anode and other reported Si-based anodes [114].

However, the combination of CNTs and silicon materials also has its limitations, which cannot effectively solve the side reactions at the surface or interface. It has been proposed that the joint use of carbon coatings and CNTs is an effective strategy to reduce side reactions and achieve high capacity of anodes [107]. Silicon nanoparticles were encapsulated in a dual carbon (C/CNTs) buffer layer formed by citric acid and ZIF-67 as precursors to improve their volume expansion [112]. It can be seen from the TEM image that the pyrolyzed ZIF-67 maintains the original polyhedral structure (Figure 9a). There are many dense CNTs on the surface, and the amorphous carbon formed by the pyrolysis of citric acid is encapsulated inside. The unique double-carbon buffer structure is conducive to the stability of SEI film. The internal amorphous carbon can partially alleviate the structural deformation, and the local flexibility of the external CNTs can reduce the stress concentration and improve structural stability. The Si@C/CNTs electrode can provide a reversible capacity of 968 mAh/g after 500 cycles at 0.5 A/g (Figure 9b,c), and a discharge capacity of 680 mAh/g after 1000 cycles at 1 A/g. The anode has a volume expansion of 47% after 300 cycles, which highlights excellent cyclic stability compared with Si@C@C (73%) and Si@C (108%). Choi et al. [113] studied the coordination of CNTs between graphite and silicon. They compared three different hybrid electrodes. Among them, the electrochemical performance of the composite (Gr-MSC) formed by adding carbon nanotubes prepared by the CVD method to the graphene/silicon-carbon composite (Gr-SC) is better than that of Gr-SC and the composite (Gr-SC + CNT) formed by adding CNTs in the slurry agitation process (Figure 9d). On the one hand, this is due to the different volume changes of silicon and graphite in the Gr-SC anode during the cycle, and the compatibility is small, which may lead to electrical contact loss. The CNTs in the Gr-MSC electrode are evenly distributed between graphite and silicon particles, which effectively maintains the electrical contact between graphite and silicon, improves the charge transfer rate, and reduces the side reactions at the interface. On the other hand, the CNTs added in the stirring make it difficult to achieve uniform distribution, and the high aspect ratio makes it easy to entangle together in the manufacturing process, causing an imbalance in the electrical pathway at the electrode level. Figure 9e,f shows the impedance comparison of the composite before and after cycling. This highlights the importance of selectivity and localization of CNTs in mixed electrodes of graphite and silicon.

Sun et al. [114] prepared a biomimetic silicon anode (AGO-Si/C) interconnected by graphene oxide (GO) sheets and CNTs (Figure 9g). Among them, the GO sheet is vertically arranged, and the silicon nanoparticles are tightly and uniformly distributed on the GO after chemisorbed and high-temperature treatment, which limits the movement of silicon nanoparticles and reduces the side reaction caused by excessive agglomeration. At the same time, this also strengthens the contact between the materials, enhances the electronic conductivity, and realizes rapid ion transport. The unique interlayer sliding and wrinkles are beneficial to alleviate the lithium stress and reduce the stress concentration. CNTs further alleviate the deformation caused by stress and strengthen the interface correlation between silicon particles and graphene oxide. The AGO-Si/C electrode has a large area capacity of 10.0 mAh/cm^2^ at a high load (4.1 mg/cm^2^). From the relaxation time distribution (DRT) diagram, it can be seen that the Rsei increases slowly from the 5th to the 100th cycle, indicating its rapid Li^+^ transfer during the cycle and its structure is very stable (Figure 9h). It is also very advantageous in comparison with other reported electrochemical properties of Si-based anodes (Figure 9i).

## 5. Other Silicon-Based Composites

### 5.1. Silicon/Conductive Polymer Composites

**Figure 10:**
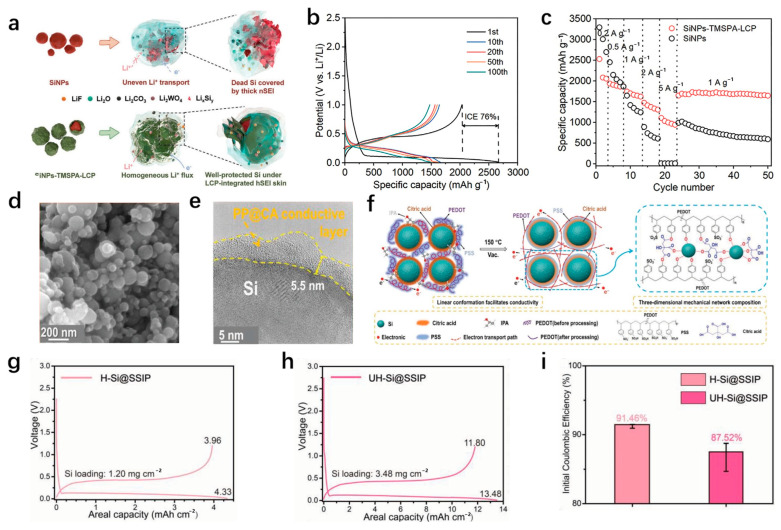
(**a**) Schematic of the evolution of interfacial stability and the SEI microstructure. (**b**) Voltage–capacity curves of SiNPs-TMSPA-LCP at 1st, 10th, 20th, 50th, and 100th. (**c**) Rate performance of SiNPs-TMSPA-LCP and Si NPs [121]. (**d**) SEM and (**e**) TEM image of Si@PP@CA. (**f**) The mechanism of functional conductive binder based on PEDOT:PSS and citric acid to enhance the stability of Si [122]. (**g**) First charging and discharging curves of H-Si@ SSIP electrode with a Si loading of 1.20 mg cm^−2^ at 0.033 C. (**h**) First charging and discharging curves of UH-Si@SSIP electrode with a Si loading of 3.84 mg cm^−2^ at 0.033 C. (**i**) ICE of H-Si@SSIP and UH-Si@SSIP electrodes [123].

In addition to the rational designs of the structures of the silicon-based anodes, the addition of conductive polymers is considered a simple and effective method to improve conductivity [115]. The traditional binder PVDF has good stability, but it has poor performance in mechanical properties and electronic and ionic conductivity. As a result, the electrode cannot provide sufficient constraints and protection for silicon nanoparticles during the cycling process, and then gradually falls off and crushes under the action of the generated stress, resulting in capacity attenuation [116]. Some conductive polymers (polyaniline [117], polypyrrole [118], polythiophene [119]) can be added to replace the insulating components to improve the electrical conductivity, and they have the adhesion of binders. Therefore, it can effectively improve some problems faced by silicon anodes. Wu et al. [120] designed a SiNPs-PANi hydrogel as an anode, and there are a large number of voids in the composite to alleviate the large structural deformation. The polyaniline coating on the surface of silicon nanoparticles can effectively provide good electrical contact and stabilize the SEI film to reduce capacity loss. The in situ polymerized SiNPs-PANi hydrogel has no obvious capacity decay after 5000 cycles at a high current density of 6 A/g, and remains at 550 mAh/g. The average Coulombic efficiency is 99.8%, showing excellent cyclic stability. Pan et al. [121] used TMSPA as a bifunctional molecular bridge to self-assemble layers of conductive polyaniline coating (LCP) on the surface of silicon nanoparticles by in situ polymerization to promote the formation of stable SEI film (Figure 10a). The directional growth of SEI film avoids overgrowth and reduces the formation of “dead lithium”. At the same time, due to the dipole–dipole interaction between ethylene fluorocarbon and LCP in the electrolyte, the components in the formed SEI film are evenly distributed, which can effectively enhance the diffusion rate and mechanical buffering performance of lithium ions. The reversible capacity is more than 1000 mAh/g after 300 cycles at a 1 A/g current, and at the current density of 5 A/g, it also has a high magnification performance of 942 mAh/g. This provides a unique insight into stabilizing the SEI film and improving the cycle stability of the silicon anode (Figure 10b,c).

Chen et al. [122] designed a Si@PP@CA anode material composed of a conductive polymer binder composed of poly (3,4-vinyl dioxythiophene), poly (styrene sulfonic acid) (PEDOT: PSS), citric acid (CA) and isopropanol (IPA) combined with silicon. Figure 10d,e showed that the Si nanoparticles were thoroughly and evenly wrapped by an outer layer with a thickness of about 5.5 nm. The conductive polymer PEDOT effectively combines with other materials through electrostatic interaction to construct a stable three-dimensional conductive network, which enhances the charge transfer rate. The CA in the periphery can quickly form a thin and solid SEI film with lithium ions to inhibit structural deformation. The proportion of silicon is as high as 90%, which has great potential in improving the energy density of silicon anode and dealing with practical applications (Figure 10f). In addition, Cai et al. [123] reported a paper on a slidable and highly ionic conductive flexible polymer binder with a specific single-ion structure (SSIP), a conductive polymer binder with high ionic conductivity. SSIPs have excellent mechanical properties, which can effectively adapt to the huge volume changes in the cycle processes and stabilize the anode structure. Strong adhesion can strengthen the connection between the components and form a stable conductive network. The internal single-ion conductive layer significantly reduces the battery impedance and improves the lithium-ion diffusion ability. The capacity of the hybrid electrode after 100, 200, and 400 cycles at 0.1 A/g, 0.2 A/g, and 0.5 A/g current densities is 2264 mAh/g, 1867 mAh/g, and 1620 mAh/g, respectively, showing excellent high-rate performance and stable cycle capacity. The Coulomb efficiencies of 91.5% and 87.5% for the first cycle of the high-area capacity (1.25 ± 0.35 mg/cm^2^) (Figure 10g) and ultra-high area capacity (3.50 ± 0.55 mg/cm^2^) (Figure 10h) silicon anodes are obtained (Figure 10i), which have great application potential in the development of stable LIBs with high energy density.

### 5.2. Silicon/Metal Composites

**Figure 11:**
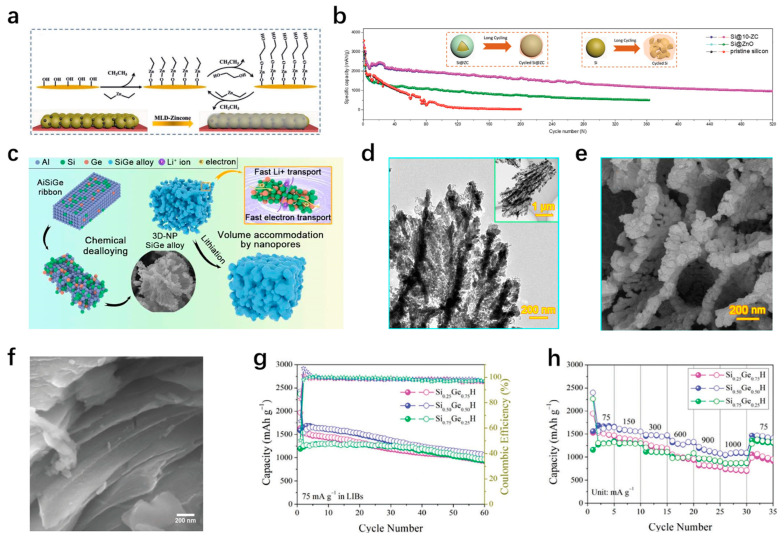
(**a**) A schematic diagram of an MLD-zincone-coated silicon electrode. (**b**) Cycling performance at 200 mAg^−1^ of Si@10-ZC [126]. (**c**) The SEM image at high magnification of Si_12_Ge_8_. (**d**) The TEM images of Si_12_Ge_8_. (**e**) Si_12_Ge_8_ material diagram [132]. Cycling performance of Si_0.25_Ge_0.75_H, Si_0.50_Ge_0.50_H, and Si_0.75_Ge_0.25_H in lithium coin cells galvanostatically cycled at a current density of 75 mA g^−1^ in terms of (**f**) specific capacity vs. cycling number. Rate capacities at current densities of 75, 150, 300, 600, 900, and 1000 mA g^−1^ in terms of (**g**) specific capacity vs. cycling number. (**h**) side-view SEM images of Si_0.50_Ge_0.50_H [133].

The combination of silicon and metal is also a strategy to effectively solve the common problems of silicon anodes. The high conductivity of metal elements can effectively improve the electrochemical performance of materials and act as a buffer material to alleviate the volume expansion of electrodes to a certain extent [124]. One approach is to coat silicon particles with metal particles to improve electrical contact. Murugesan et al. [125] used the polyol method to uniformly deposit Cu on hydrogenated amorphous silicon particles to form a composite (Cu-coated A-Si: H). The Cu particles deposited on the surface can reduce the contact loss between particles caused by the formation of SEI, and the conductive network formed is conducive to the diffusion of lithium ions in silicon particles, and obtain higher cyclic stability. Mu et al. [126] uniformly and controllably deposited a layer of zinc coating on the silicon anode (Figure 11a). The presence of metallic zinc accelerates the charge transfer and promotes faster reaction kinetics. In the formation of SEI film, a stable and efficient interface with good ionic and electronic conductivities is formed due to the effective coordination of zinc and organic polymer. The reversible capacity of the hybrid electrode is 1741 mAh/g after 100 cycles at 200 mA/g (Figure 11b), and it also has excellent performance in rate performance. The reversible capacities at 2 A/g and 5 A/g are 1580 mAh/g and 970 mAh/g, respectively, which indicates that zinc coating (≈3 nm) greatly improves the cycling performance and rate capacity of silicon anode. The addition of metal particles can also improve the interface reaction between silicon and carbon materials, and further improve the performance of silicon anodes. Li et al. [127] introduced Bi nanoparticles on the surface of silicon particles and used them as a fast transport bridge for lithium ions after pre-lithium. The peripheral N-doped C could alleviate the volume change in the Si/Bi electrode during cycling. The unique structure has a high reversible capacity of 477.8 mAh/g at a high current density of 10 A/g.

In addition, alloy anodes formed by alloying is also a good choice [128]. The combination of metal and silicon can also be formed by alloying, and the strong mechanical properties of the silicon metal alloy can ensure its structural integrity during the cycle [129]. Among the many alternative metals, the metal Ge has high conductivity and lithium-ion diffusion rate [130,131], and the combination with silicon can effectively exert the advantages of both. Yang et al. [132] synthesized a coral-like SiGe alloy by dealloying the AlSiGe strip alloy (Figure 11c). Its surface has abundant micropores and mesoporous, which increases the specific surface area of the material and provides more active sites. It can be seen from the electron microscope that the synthesized material is dendritic and there are various channels between dendrites, which may be related to the growth of dealloyed SiGe alloy. The formed channels are conducive to the transport of Li^+^ and provide buffer space for the volume expansion during the cycle (Figure 11d,e). Meanwhile, the pore distribution of the synthesized SiGe alloy could be adjusted by adjusting the content of Al in the precursor. Among them, the Si_12_Ge_8_ (Al content is 80%) alloy exhibits the best electrochemical performance, and its capacity remains at 1158 mAh/g after 1000 cycles at a current density of 1 A/g. This is attributed to the unique coral-like structure and the synergistic effect of Ge, which makes the electrode have the most excellent lithium ion transport kinetics and provides an alternative method for large-scale production of electrodes with predictable and controllable nanostructures. Wei et al. [133] prepared Si_x_Ge_1−x_H composites by the chemical etching process. Figure 11f shows the topography of the material. Due to the HF etching, the defect sites of the composites are increased, which improves the conductivity. At the same time, the loose layered structure of the amorphous composite enhances mechanical flexibility, which is conducive to cycling stability. By testing the electrochemical properties of different ratios (x is 0.25, 0.5, 0.75), it is found that Si_0.5_Ge_0.5_H forms the most favorable void structure, which is conducive to the penetration of electrolytes and the transport of lithium ions in the deintercalated lithium. It has better electrochemical performance than the alloys synthesized in the other two ratios (Figure 11g,h). Table 2 shows the electrochemical properties of some of the silicon-based composite materials mentioned above.

## 6. Summary and Prospect

This paper introduces the research progress of silicon-based composites as anode materials for lithium-ion batteries in recent years. As a highly concerned lithium anode material, silicon has a very high theoretical capacity and has been considered one of the substitutes for the next generation of lithium-ion battery anode materials. However, the problem of large volume change and poor conductivity during the cycle has been criticized. Designing it into a reasonable nanostructure or compounding it with other materials can effectively improve these problems. For example, the core–shell structure provides a buffer space for the volume change of silicon, which can effectively suppress the volume expansion during the cycle and reduce the capacity loss. The hollow porous structure can accelerate the diffusion rate of ions and reduce the diffusion distance. As one-dimensional structures, silicon nanowires and nanotubes can effectively alleviate the stress and strain during cycling. The silicon-based 3D network constructed by graphene or 3D printing provides an effective path for electron conduction and maintains the integrity of the negative electrode structure. At the same time, compounding with carbon materials or metal materials can improve the conductivity of the composite material and effectively improve its electrochemical performance.

Some nanostructured silicon-based anode materials are often designed to improve cycle performance at the expense of their volume energy density, and the preparation process is usually cumbersome, which is contrary to commercial standards. Therefore, the future development of silicon-based anodes can start from the following aspects. Firstly, the contribution of silicon content in the composite material to the capacity is very important, and increasing the proportion of silicon will cause serious volume expansion problems. When silicon is compounded with other materials, it is often necessary to consider the silicon content to find a balance between affecting its capacity and cycle performance. Secondly, the development of suitable binders and electrolytes, the doping of various elements or heteroatoms such as N, P, S, etc., is crucial to the improvement of the electrochemical performance of silicon-based electrode materials. This will help to form a stable SEI film during the cycle and improve the cycle life of the electrode. Thirdly, it is necessary to explore a new pre-lithiation technology to improve the first coulombic efficiency of silicon-based anode materials, while reducing the cost of pre-lithiation technology, which is convenient for large-scale production, and the stability of pre-lithium electrodes in air needs to be solved as soon as possible. Finally, the micron structure has a higher volume energy density than the nanostructure, but it also lacks the advantages of the nanostructure. Therefore, it is a feasible strategy to consider one or more properties of nanostructures on micron-sized silicon-based materials.

Although silicon has a very attractive theoretical capacity, its full adoption in the market is still not a small gap. This is not only reflected in the defects of silicon itself, but also in some external factors, such as low cost, mass production, simple experimental procedures, and environmental friendliness. Therefore, when designing experimental steps, it is necessary to consider whether all indicators are taken into account. At present, there have been a lot of experimenters in this area of research, they are gradually solving the obstacles on the road to commercialization of silicon anodes. Therefore, we believe that high-energy density silicon anode lithium-ion batteries will be developed and recognized by most people in the near future.

## Figures and Tables

**Table 1:** Electrochemical properties of several nanostructured silicon anodes have been reported.

Anode	Current Density	1st Discharge Capacity	ICE	Cycling Performance	Rate Performance	The Mass Loading	Ref.
Si@NSC	300 mA/g	1906 mAh/g	82.00%	1720 mAh/g /550 cycle	235 mAh/g /4 A/g	1–2 mg/cm^2^	[43]
Si@V_3_O_4_@C	500 mA/g	1517 mAh/g	/	1061 mAh/g /700 cycle	978 mAh/g /2 A/g	0.5 mg/cm^2^	[44]
H-SiGe/pC	100 mA/g (the initial one cycles) /4 A/g	2922 mAh/g	83.10%	1215.6 mAh/g /200 cycle	1255.4 mAh/g /8 A/g	1–1.2 mg/cm^2^	[50]
N-PoSi@C	100 mA/g	2747 mAh/g	85%	1645.6 mAh/g /200 cycle	904.7 mAh/g/12.8 A/g	1 mg/cm^2^	[56]
NPSi@C	200 mA/g	1306.9 mAh/g	41%	681.8 mAh/g /100 cycle	294.2 mAh/g/2 A/g	1 mg/cm^2^	[58]
G/SiNW@CNT/cPAN	200 mA/g (the initial five cycles) /1 A/g	~2500 mAh/g	82.31%	1296 mAh/g /200 cycle	886 mAh/g /8 A/g	1.2 mg/cm^2^	[66]
Si NWs	100 mA/g	3447.8 mAh/g	/	2410 mAh/g /60 cycle	306 mAh/g /2 A/g	/	[67]
SiNTs	0.05 C (1 C = 4.2 A/g)	4950 mAh/g	63.00%	1670 mAh/g /30 cycle	450 mAh/g /1 C	~0.5 mg/cm^2^	[82]
Si-NT/Graphite	100 mA/g	3348 mAh/g	88.80%	1579 mAh/g /200 cycle (1 A/g)	472 mAh/g /5 A/g	/	[74]
SNT	100 mA/g	3442 mAh/g	89%	2045 mAh/g /200 cycle (0.5 A/g)	1427 mAh/g /2 A/g	1 mg/cm^2^	[75]
GSSSE	239 mA/g	2599 mAh/g	71.90%	1314 mAh/g /500 cycle (797 mAh/g)	301 mAh/g /28,680 mA/g	0.13 mg/cm^2^	[80]
D_SiAg_3_	0.2 C	2850 mAh/g	93%	~1750 mAh/g /100 cycle (1 C)	1250 mAh/g /10 C	/	[83]

**Table 2:** Electrochemical properties of carbon, conductive polymer, metal, and silicon composites of some materials.

Anode	Current Density	1st Discharge Capacity	ICE	Cycling Performance	Rate Performance	The Mass Loading	Ref.
LESG	0.1 A/g	502 mAh/g	116%	620 mAh/g /8 cycle	/	3.5 mg/cm^2^	[97]
W-120	0.1 A/g	~1100 mAh/g	72.50%	632 mAh/g /100 cycle	182 mAh/g /2 A/g	3.9 mg/cm^2^	[98]
NG@SiC	0.1 A/g	1496.8 mAh/g	85.80%	1197.5 mAh/g /200 cycle	437.1 mAh/g /10 A/g	/	[105]
SiNP/LSG	0.2 A/g	1074 mAh/g	/	88.3%/100 cycle /2 A/g	335 mAh/g /10 A/g	0.2 mg/cm^2^	[106]
Si@C@CNTs	0.5 A/g	1100 mAh/g	/	968 mAh/g /500 cycle	861 mAh/g /3 A/g	1.3 mg/cm^2^	[112]
Gr-MSC	0.1 C	1453 mAh/g	80.50%	98.4%/50 cycle /0.5 C	/	0.7 mg/cm^2^	[113]
Si@PP@CA	0.2 A/g	2875 mAh/g	/	2530 mAh/g /100 cycle	1785 mAh/g /1 A/g	0.2–0.4 mg/cm^2^	[122]
HSi@SSIP	0.033 C	13.48 mAh/cm^2^ (1500 mAh/g = 1.85 Ah/cm^2^)	91.50%	2.10 mAh/cm^2^ /100 cycle/0.2 C	/	1.25 ± 0.35 mg/cm^2^	[123]
Si/Bi@NC	0.1 A/g	1939.2 mAh/g	71.50%	876.7 mAh/g /450 cycle/1 A/g	477.8 mAh/g /10 A/g	0.5 mg/cm^2^	[127]
Si0.5Ge0.5	0.075 A/g	2419 mAh/g	66.00%	1059 mAh/g /60 cycle	1106 mAh/g /1 A/g	0.9 mg/cm^2^– 1.2 mg/cm^2^	[133]

## Data Availability

No new data were created or analyzed in this study. Data sharing is not applicable to this article.

## References

[1] Lashof D.A., Ahuja D.R. (1990). Relative Contributions of greenhouse gas emissions to global warming. Nature.

[2] Rodhe H. (1990). A comparison of the contribution of various gases to the greenhouse effect. Science.

[3] Duffy P.B., Field C.B., Diffenbaugh N.S., Doney S.C., Dutton Z., Goodman S., Heinzerling L., Hsiang S., Lobell D.B., Mickley L.J. (2019). Strengthened scientific support for the endangerment finding for atmospheric greenhouse gases. Science.

[4] Hoegh-Guldberg O., Bruno J.F. (2010). The impact of climate change on the world’s marine ecosystems. Science.

[5] Aramendia E., Brockway P.E., Taylor P.G., Norman J.B., Heun M.K., Marshall Z. (2024). Estimation of useful-stage energy returns on investment for fossil fuels and implications for renewable energy systems. Nat. Energy.

[6] Lei Y.D., Wang Z.L., Wang D.Y., Zhang X.Y., Che H.Z., Yue X., Tian C.G., Zhong J.T., Guo L.F., Li L. (2023). Co-benefits of carbon neutrality in enhancing and stabilizing solar and wind energy. Nat. Clim. Change.

[7] Arbabzadeh M., Sioshansi R., Johnson J.X., Keoleian G.A. (2019). The role of energy storage in deep decarbonization of electricity production. Nat. Commun..

[8] Citroni R., Mangini F., Frezza F. (2024). Efficient Integration of Ultra-low Power Techniques and Energy Harvesting in Self-Sufficient Devices: A Comprehensive Overview of Current Progress and Future Directions. Sensors.

[9] Armand M., Tarascon J.M. (2008). Building better batteries. Nature.

[10] Scrosati B., Garche J. (2010). Lithium batteries: Status, prospects and future. J. Power Sources.

[11] Dai X.Q., Liu H.T., Liu X., Liu Z.L., Liu Y.S., Cao Y.H., Tao J.Y., Shan Z.Q. (2021). Silicon nanoparticles encapsulated in multifunctional crosslinked nano-silica/carbon hybrid matrix as a high-performance anode for Li-ion batteries. Chem. Eng. J..

[12] Aricò A.S., Bruce P., Scrosati B., Tarascon J.M., Schalkwijk W. (2005). Nanostructured materials for advanced energy conversion and storage devices. Nat. Mater..

[13] Li W.W., Ma Q.B., Shen P.F., Zhou Y.C., Soule L.K., Li Y.Y., Wu Y.X., Zhang H.Y., Liu M.L. (2021). Yolk-shell structured CuSi_2_P_3_@Graphene nanocomposite anode for long-life and high-rate lithium-ion batteries. Nano Energy.

[14] Jiao X.X., Liu Y.Y., Li B., Zhang W.X., He C., Zhang C.F., Yu Z.X., Gao T.Y., Song J.X. (2019). Amorphous phosphorus-carbon nanotube hybrid anode with ultralong cycle life and high-rate capability for lithium-ion battery. Carbon.

[15] Baek K., Lee W.G., Im E., Ha J.H., Ahn S., Kim Y., Choi Y., Kang S.J. (2023). Gradient lithium metal infusion in Ag-decorated carbon fibers for High-Capacity lithium metal battery anodes. Nano Lett..

[16] Yuan X.L., Ma Z.T., Jian S.F., Ma H., Lai Y.A., Deng S.L., Tian X.C., Wong C.P., Xia F., Dong Y.F. (2022). Mesoporous nitrogen-doped carbon MnO_2_ multichannel nanotubes with high performance for Li-ion batteries. Nano Energy.

[17] Zou B.B., Wang T., Li S.Y., Kang R., Li G.C., EI-Khodary S.A., Ng D.H.L., Liu X.H., Qiu J.X., Zhao Y. (2021). In situ XRD and electrochemical investigation on a new intercalation-type anode for high-rate lithium ion capacitor. J. Energy Chem..

[18] Yu S.H., Lee S.H., Lee D.J., Sung Y.E., Hyeon T. (2015). Conversion Reaction-Based Oxide Nanomaterials for Lithium Ion Battery Anodes. Small.

[19] Peng M.Q., Shin K., Jiang L.X., Jin Y., Zeng K., Zhou X.L., Tang Y.B. (2022). Alloy-type anodes for high-performance rechargeable batteries. Angew. Chem. Int. Edit..

[20] Yao Y.X., Yan C., Zhang Q. (2020). Emerging interfacial chemistry of graphite anodes in lithium-ion batteries. Chem. Commun..

[21] Song X.Y., Kinoshita K., Tran T.D. (1996). Electrochem. Microstructural Characterization of Lithiated Graphite. J. Electrochem. Soc..

[22] Wang Y., Roller J., Maric R. (2018). Novel flame synthesis of nanostructured α-Fe_2_O_3_ electrode as high-performance anode for lithium ion batteries. J. Power Sources.

[23] Zhou Z., Ding C.Y., Peng W.C., Li Y., Zhang F.B., Fan X.B. (2021). One-step fabrication of two-dimensional hierarchical Mn_2_O_3_@graphene composite as high-performance anode materials for lithium ion batteries. J. Mater. Sci. Technol..

[24] Lou Y.B., He D., Wang Z.F., Hu Y.H., Shen Y., Ming J., Chen J.X. (2017). Nanocomposite of ultrasmall Co_3_O_4_ nanoparticles deposited on ultrathin MoS_2_ surfaces for excellent performance anode materials in lithium ion batteries. Chem. Eng. J..

[25] Sahu S.R., Rikka V.R., Haridoss P., Chatterjee A., Gopalan R., Prakash R. (2020). A novel α-MoO_3_/single-walled carbon nanohorns composite as high-performance anode material for fast-charging iithium-ion battery. Adv. Energy Mater..

[26] Hassan F.M., Batmaz R., Li J.D., Wang X.L., Xiao X.C., Yu A.P., Chen Z.W. (2015). Evidence of covalent synergy in silicon–sulfur–graphene yielding highly efficient and long-life lithium-ion batteries. Nat. Commun..

[27] Wang H., Song W., Liu M.F., Zhang S.Y., Ren L., Qiu D., Chen X.Q., Yang K. (2022). Manufacture-friendly nanostructured metalsstabilized by dual-phase honeycomb shell. Nat. Commun..

[28] Liu N., Hu L.B., McDowell M.T., Jackson A., Cui Y. (2011). Prelithiated silicon nanowires as an anode for lithium ion batteries. ACS Nano.

[29] Guo S.C., Hu X., Hou Y., Wen Z.H. (2017). Tunable synthesis of yolk-shell porous silicon@carbon for optimizing Si/C-based anode of lithium-ion batteries. ACS Appl. Mater. Interfaces.

[30] Bogart T.D., Oka D., Lu X.T., Gu M., Wang C.M., Korgel B.A. (2014). Lithium ion battery peformance of silicon nanowires with carbon skin. ACS Nano.

[31] Bitew Z., Tesemma M., Beyene Y., Amare M. (2022). Nano structured silicon and silicon based composites as anode materials for lithium ion batteries: Recent progress and perspectives. Sustain. Energy Fuels.

[32] Xiao Z.L., Wang C., Song L.B., Zheng Y.H., Long T.Y. (2022). Research progress of nano-silicon-based materials and silicon-carbon composite anode materials for lithium-ion batteries. J. Solid State Electr..

[33] Xu L.X. (2023). Analysis of research progress in improving the performance of silicon-based anode materials for lithium-ion batteries: A combination of nanostructured silicon and silicon-based composites. J. Phys. Conf. Ser..

[34] Jain R., Lakhnot A.S., Bhimani K., Sharma S., Mahajani V., Panchal R.A., Kamble M., Han F.D., Wang C.S., Koratkar N. (2022). Nanostructuring versus microstructuring in battery electrodes. Nat. Rev. Mater..

[35] Liu X.H., Zhong L., Huang S., Mao S.X., Zhu T., Huang J.Y. (2012). Size-dependent fracture of silicon nanoparticles during lithiation. ACS Nano.

[36] Zhang Q.F., Uchaker E., Candelaria S.L., Cao G.Z. (2013). Nanomaterials for energy conversion and storage. Chem. Soc. Rev..

[37] Wen C.J., Huggins R.A. (1981). Chemical diffusion in intermediate phases in the lithium-silicon system. J. Solid State Chem..

[38] Limthongkul P., Jang Y., Dudney N.J., Chiang Y.M. (2003). Electrochemically-driven solid-state amorphization in lithium-silicon alloys and implications for lithium storage. Acta Mater..

[39] McDowell M.T., Lee S.W., Nix W.D., Cui Y. (2013). 25th anniversary article: Understanding the lithiation of silicon and other alloying anodes for lithium-ion batteries. Adv. Mater..

[40] Prakash S., Zhang C.F., Park J.D., Razmjooei F., Yu J.S. (2019). Silicon core-mesoporous shell carbon spheres as high stability lithium-ion battery anode. J. Colloid Interf. Sci..

[41] Lu W.J., Guo X.T., Luo Y.Q., Li Q., Zhu R.M., Pang H. (2019). Core-shell materials for advanced batteries. Chem. Eng. J..

[42] Li J.B., Fan S., Xiu H.J., Wu H.W., Huang S.Y., Wang S.M., Yin D.W., Deng Z.L., Xiong C.Y. (2023). TiO_2_-coated silicon nanoparticle core-shell structure for high-capacity lithium-ion battery anode materials. Nanomaterials.

[43] He Q., Wu Q.Y., Wang X.X., Fu S.T., Huang S.C., Tong S.F., Cao Y.F., Liu Z., Wu M.M. (2021). An anode material for lithium storage: Si@N,S-doped carbon synthesized via in situ self-polymerization. ACS Appl. Energy Mater..

[44] Wang Z.Y., Yao M., Luo H., Xu C.H.Y., Tian H., Wang Q., Wu H., Zhang Q.Y., Wu Y.P. (2024). Rational design of ion-conductive layer on Si anode enables superior-stable lithium-ion batteries. Small.

[45] Lei Y., Li S., Du M., Mi J., Gao D.C., Hao L., Jiang L.J., Man L., Jiang W.Q., Li F. (2023). Preparation of double-shell Si@SnO_2_@C nanocomposite as anode for lithium-ion batteries by hydrothermal method. Rare Metals.

[46] Zhang R.S., Jia F.D., Sun C.X., Pan J.H., Wang F.F., Sang J.J., Gao C., Li S.L., Wang Q. (2024). Enhanced lithium storage performance: Dual-modified electrospun Si@MnO@CNFs composites for advanced anodes. ACS Appl. Mater. Interfaces.

[47] Yoon T., Bok T., Kim C., Na Y., Park S., Kim K.S. (2017). Mesoporous silicon hollow nanocubes derived from metal-organic framework template for advanced lithium-ion battery anode. ACS Nano.

[48] Lu B., Ma B.J., Deng X.L., Wu B., Wu Z.Y., Luo J., Wang X.Y., Chen G.R. (2018). Dual stabilized architecture of hollow Si@TiO_2_@C nanospheres as anode of high-performance Li-ion battery. Chem. Eng. J..

[49] Park Y., Choi N.S., Park S., Woo S.H., Sim S.J., Jang B.Y., Oh S.M., Park S., Cho J., Lee K.T. (2013). Si-encapsulating hollow carbon electrodes via electroless etching for lithium-ion batteries. Adv. Energy Mater..

[50] Gao P.B., Wu H.M., Liu W.H., Tian S., Mu J.L., Miao Z.C., Zhou P.F., Zhang H.N., Zhou T., Zhou J. (2023). Heterogeneousisomorphism hollow SiGe nanospheres with porous carbon reinforcing for superior electrochemical lithium storage. J. Energy Chem..

[51] Gao J.F., Zuo S.L., Liu H., Jiang Q.W., Wang C.H., Yin H.H., Wang Z.Q., Wang J. (2022). An interconnected and scalable hollow Si-C nanospheres/graphite composite for high-performance lithium-ion batteries. J. Colloid Interf. Sci..

[52] He Z.Y., Liu L., Liu S.N., Chen Y., Sun L., Liu C., Zhu Y.C., Wang X.F. (2023). A novel design idea of high-stability silicon anodes for lithium-ion batteries: Building in-situ “high-speed channels” while reserving space. Chem. Eng. J..

[53] Tao Y., Tian Y., An Y.L., Wei C.L., Li Y., Zhang Q.K., Feng J.K. (2021). Green and facile fabrication of nanoporous silicon@carbon from commercial alloy with high graphitization degree for high-energy lithium-ion batteries. Sustain. Mater. Techno..

[54] Ren Y., Yin X.C., Xiao R., Mu T.S., Huo H., Zuo P.J., Ma Y.L., Cheng X.Q., Gao Y.Z., Yin G.P. (2022). Layered porous silicon encapsulated in carbon nanotube cage as ultra-stable anode for lithium-ion batteries. Chem. Eng. J..

[55] McDowell M.T., Ryu I., Lee S.W., Wang C.M., Nix W.D., Cui Y. (2012). Studying the kinetics of crystalline silicon nanoparticle lithiation with in situ transmission electron microscopy. Adv. Mater..

[56] Yan Z., Jiang J., Zhang Y., Yang D., Du N. (2022). Scalable and low-cost synthesis of porous silicon nanoparticles as high-performance lithium-ion battery anode. Mater. Today Nano.

[57] Wada T., Ichitsubo T., Yubuta K., Segawa H., Yoshida H., Kato H. (2014). Bulk-nanoporous-silicon negative electrode with extremely high cyclability for lithium-ion batteries prepared using a top-down process. Nano Lett..

[58] Wang Z.G., Zheng B., Liu H., Zhang C., Wu F.F., Luo H.Y., Yu P. (2021). One-step synthesis of nanoporous silicon @ graphitized carbon composite and its superior lithium storage properties. J. Alloy. Compd..

[59] Chan C.K., Peng H.L., Liu G., Mcllwrath K., Zhang X.F., Huggins R.A., Cui Y. (2008). High-performance lithium battery anodes using silicon nanowires. Nat. Nanotechnol..

[60] Tu J.X., Yu S., Hao K., Sun L., Bai R.C., Zhang F.Z., Li A.J., Liu H. (2024). Controllable synthesis of one-dimensional silicon nanostructures based on the dual effects of electro-deoxidation and the Kirkendall effect. Nano Res..

[61] Amiinu I.S., Imtiaz S., Geaney H., Kennedy T., Kapuria N., Singh S., Ryan K.M. (2023). A thin Si nanowire network anode for high volumetric capacity and long-life lithium-ion batteries. J. Energy Chem..

[62] Nguyen H.T., Yao F., Zamfir M.R., Biswas C., So K.P., Lee Y.H., Kim S.M., Cha S.N., Kim J.M., Pribat D. (2011). Highly interconnected Si nanowires for improved stability Li-ion battery anodes. Adv. Energy Mater..

[63] Aminu I.S., Geaney H., Imtiaz S., Adegoke T.E., Kapuria N., Collins G.A., Ryan K.M. (2020). A copper silicide nanofoam current collector for directly grown Si nanowire networks and their application as lithium-ion anodes. Adv. Funct. Mater..

[64] Xia F., Kwon S., Lee W.W., Liu Z.M., Kim S., Song T., Choi K.J., Paik U., Park W.I. (2015). Graphene as an interfacial layer for improving cycling performance of Si nanowires in lithium-ion batteries. Nano Lett..

[65] Stokes K., Flynn G., Geaney H., Bree G., Ryan K.M. (2018). Axial Si-Ge heterostructure nanowires as lithium-ion battery anodes. Nano Lett..

[66] Li F.H., Wu H., Wen H., Wang C., Shen C.Q., Su L.W., Liu S., Chen Y.F., Wang L.B. (2024). Constructing a stable integrated silicon electrode with efficient lithium storage performance through multidimensional structural design. ACS Appl. Mater. Interfaces.

[67] Pang Z.Y., Xiong X.L., Tian F., Zhang X.Q., Wang F., Xia X.W., Li G.S., Sun C.T., Wang S.J., Yu X. (2024). A sustainable molten salt leaching-electrodeposition route toward converting rice husks into functional silicon nanowires and porous carbon. ACS Sustainable Chem. Eng..

[68] Wang F., Li P., Li W., Wang D.H. (2022). Electrochemical synthesis of multidimensional nanostructured silicon as a negative electrode material for lithium-ion battery. ACS Nano.

[69] Park M.H., Kim M.G., Joo J., Kim K., Kim J., Ahn S., Cui Y., Cho J. (2009). Silicon nanotube battery anodes. Nano Lett..

[70] Liu J.Y., Li N., Goodman M.D., Zhang H.G., Epstein E.S., Huang B., Pan Z., Kim J., Choi J.H., Huang X.J. (2015). Mechanically and chemically robust sandwich-structured C@Si@C nanotube array Li-ion battery anodes. ACS Nano.

[71] Song T., Xia J.L., Lee J.H., Lee D.H., Kwon M.S., Choi J.M., Wu J., Doo S.K., Chang H., Park W.I. (2010). Arrays of sealed silicon nanotubes as anodes for lithium ion batteries. Nano Lett..

[72] Tesfaye A.T., Gonzalez R., Coffer J.L., Djenizian T. (2015). Porous silicon nanotube arrays as anode material for Li-ion batteries. ACS Appl. Mater. Interfaces.

[73] Wang W., Gu L., Qian H.L., Zhao M., Ding X., Peng X.S., Sha J., Wang Y.W. (2016). Carbon-coated silicon nanotube arrays on carbon cloth as a hybrid anode for lithium-ion batteries. J. Power Sources.

[74] Jing S.X., Xiao J.X., Shen Y.J., Hong B., Gu D., Xiao W. (2022). Silicate-mediated electrolytic silicon nanotube from silica in molten salts. Small.

[75] Wang F., Liu W., Li P., Guan Z.H., Li W. (2024). Wang, D.H. Self-assembly of silicon nanotubes driven by a biphasic transition from the natural mineral montmorillonite in molten salt electrolysis. Small.

[76] Elomari G., Hdidou L., Larhlimi H., Aqil M., Makha M., Alami J., Dahbi M. (2024). Sputtered silicon-coated graphite electrodes as high cycling stability and improved kinetics anodes for lithium ion batteries. ACS Appl. Mater. Interfaces.

[77] Kasavajjula U., Wang C., Appleby A.J. (2007). Nano- and bulk-silicon-based insertion anodes for lithium-ion secondary cells. J. Power Sources.

[78] Soni S.K., Sheldon B.W., Xiao X.C., Tokranov A. (2011). Thickness effects on the lithiation of amorphous silicon thin films. Scripta Mater..

[79] Shen T., Yao Z.J., Xia X.H., Wang X.L., Gu C.D., Tu J.P. (2018). Rationally designed silicon nanostructures as anode material for lithium-ion batteries. Adv. Eng. Mater..

[80] Wang C.D., Chui Y.S., Ma R., Wong T., Ren J.G., Wu Q.H., Chen X.F., Zhang W.J. (2013). A three-dimensional graphene scaffold supported thin film silicon anode for lithium-ion batteries. J. Mater. Chem. A.

[81] Yu C.J., Li X., Ma T., Rong J.P., Zhang R.J., Shaffer J., An Y.H., Liu Q., Wei B.Q., Jiang H.Q. (2012). Silicon thin films as anodes for high-performance lithium-ion batteries with effective stress relaxation. Adv. Energy Mater..

[82] Suresh S., Wu Z.P., Bartolucci S.F., Basu S., Mukherjee R., Gupta T., Hundekar P., Shi Y.F., Lu T.M., Koratkar N. (2017). Protecting silicon film anodes in lithium-ion batteries using an atomically thin graphene drape. ACS Nano.

[83] Chen Y.X., Liao H.C., Cheng Y.W., Huang J.H., Liu C.P. (2023). Scalable interlayer nanostructure design for high-rate (10C) submicron silicon-film electrode by incorporating silver nanoparticles. ACS Appl. Mater. Interfaces.

[84] Xi F.S., Zhang Z., Hu Y.X., Li S.Y., Ma W.H., Chen X.H., Wan X.H., Chong C.M., Luo B., Wang L.Z. (2021). PSi@SiO_x_/Nano-Ag composite derived from silicon cutting waste as high-performance anode material for Li-ion batteries. J. Hazard. Mater..

[85] Adhitama E., Wickeren S.V., Neuhaus K., Frankenstein L., Demelash F., Javed A., Haneke L., Nowak S., Winter M., Martin A.G. (2022). Revealing the role, mechanism, and impact of AlF_3_ coatings on the interphase of silicon thin film anodes. Adv. Energy Materi..

[86] Shen X.H., Tian Z.Y., Fan R.J., Shao L., Zhang D.P., Cao G.L., Kou L., Bai Y.Z. (2018). Research progress on silicon/carbon composite anode materials for lithium-ion battery. J. Energy Chem..

[87] Kim N., Chae S., Ma J., Ko M., Cho J. (2017). Fast-charging high-energy lithium-ion batteries via implantation of amorphous silicon nanolayer in edge-plane activated graphite anodes. Nat. Commun..

[88] Son I.H., Park J.H., Kwon S., Park S., Rümmeli M.H., Bachmatiuk A., Song H.J., Ku J., Choi J.W., Choi J.W. (2015). Silicon carbide-free graphene growth on silicon for lithium-ion battery with high volumetric energy density. Nat. Commun..

[89] Xiao Q.Z., Fan Y., Wang X.H., Susantyoko R.A., Zhang Q. (2014). A multilayer Si/CNT coaxial nanofiber LIB anode with a high areal capacity. Energy Environ. Sci..

[90] Ma Q., Dai Y., Wang H.R., Ma G.Z., Guo H., Zeng X.X., Tu N.M., Wu X.W., Xiao M.T. (2021). Directly conversion the biomass-waste to Si/C composite anode materials for advanced lithium ion batteries. Chin. Chem. Lett..

[91] Gao R.S., Tang J., Yu X.L., Tang S., Ozawa K., Sasaki T., Qin L.C. (2020). In situ synthesis of MOF-derived carbon shells for silicon anode with improved lithium-ion storage. Nano Energy.

[92] Peng Q., Rehman J., Eid K., Alofi A.S., Laref A., Albaqami M.D., Alotabi R.G., Shibl M.F. (2022). Vanadium Carbide (V_4_C_3_) MXene as an Efficient Anode for Li-Ion and Na-Ion Batteries. Nanomaterials.

[93] Nitta N., Wu F.X., Lee J.T., Yushin G. (2015). Li-ion battery materials: Present and future. Mater. Today.

[94] Li M., Hou X.H., Sha Y.J., Wang J., Hu S.J., Liu X., Shao Z.P. (2014). Facile spray-drying/pyrolysis synthesis of core- shell structure graphite/silicon-porous carbon composite as a superior anode for Li-ion batteries. J. Power Sources.

[95] Yan Z.L., Yi S., Li X.D., Jiang J.W., Yang D.R., Du N. (2023). A scalable silicon/graphite anode with high silicon content for high-energy lithium-ion batteries. Mater. Today Energy.

[96] Berhaut C.L., Dominguez D.Z., Tomasi D., Vincens C., Haon C., Reynier Y., Porcher W., Boudet N., Blanc N., Chahine G.A. (2020). Prelithiation of silicon/graphite composite anodes: Benefits and mechanisms for long-lasting Li-ion batteries. Energy Storage Mater..

[97] Gao Y.J., Cui C.H., Huang Z.K., Pan G.Y., Gu Y.F., Yang Y.N., Bai F., Sun Z., Zhang T. (2024). Lithium pre-storage enables high initial coulombic efficiency and stable lithium-enriched silicon/graphite anode, Angew. Chem. Int. Edit..

[98] Lai W.H., Lee J.H., Shi L., Liu Y.Q., Pu Y.H., Ong Y.K., Limpo C., Xiong T., Rao Y.F., Sow C.H. (2024). High mechanical strength Si anode synthesis with interlayer bonded expanded graphite structure for lithium-ion batteries. J. Energy Chem..

[99] Raccichini R., Varzi A., Passerini S., Scrosati B. (2015). The role of graphene for electrochemical energy storage. Nat. Mater..

[100] Ambrosi A., Chua C.K., Latiff N.M., Loo A.H., Wong C.H.A., Eng A.Y.S., Bonanni A., Pumera M. (2016). Graphene and its electrochemistry—An update. Chem. Soc. Rev..

[101] Zhu Y., James D.K., Tour J.M. (2012). New routes to graphene, graphene oxide and their related applications. Adv. Mater..

[102] Li N., Jin S.X., Liao Q.Y., Cui H., Wang C.X. (2014). Encapsulated within graphene shell silicon nanoparticles anchored on vertically aligned graphene trees as lithium ion battery anodes. Nano Energy.

[103] Park H.I., Park Y.K., Kim S.K., Jang H.D., Kim H. (2021). Hollow graphene as an expansion-inhibiting electrical interconnector for silicon electrodes in lithium-ion batteries. ACS Appl. Mater. Interfaces.

[104] Ma Z.H., Wang L.J., Wang D.D., Huang R.H., Wang C.J., Chen G.R., Miao C.Q., Peng Y.J., Li A.Q., Miao Y. (2022). Crucial contact interface of Si@graphene anodes for high-performance Li-ion batteries. Appl. Surf. Sci..

[105] Sun C.L., Xu X., Gui C.L., Chen F.Z., Wang Y.A., Chen S.Z., Shao M.H., Wang J.H. (2023). High-quality epitaxial N doped graphene on SiC with tunable interfacial interactions via electron/ion bridges for stable lithium-ion storage. Nano-Micro Lett..

[106] Katsuyama Y., Yang Z.Y., Thiel M., Zhang X.Y., Chang X.Y., Lin C.W., Huang A.L., Wang C.X., Li Y.Z., Kaner R.B. (2024). A rapid, scalable laser-scribing process to prepare Si/graphene composites for lithium-ion batteries. Small.

[107] He Z.Y., Zhang C.X., Zhu Z.X., Yu Y.X., Zheng C., Wei F. (2024). Advances in carbon nanotubes and carbon coatings as conductive networks in silicon-based anodes. Adv. Funct. Mater..

[108] De Volder M.F.L., Tawfick S.H., Baughman R.H., Hart A.J. (2013). Carbon nanotubes: Present and future commercial applications. Science.

[109] Jin D., Yang X.F., Ou Y.Q., Rao M.M., Zhong Y.T., Zhou G.M., Ye D.Q., Qiu Y.C., Wu Y.P., Li W.S. (2020). Thermal pyrolysis of Si@ZIF-67 into Si@N-doped CNTs towards highly stable lithium storage. Sci. Bull..

[110] Lee B.S., Yoon J., Jung C., Kim D.Y., Jeon S.Y., Kim K.H., Park J.H., Park H., Lee K.H., Kang Y.S. (2016). Silicon/carbon nanotube/BaTiO_3_ nanocomposite anode: Evidence for enhanced lithium-ion mobility induced by the local piezoelectric potential. ACS Nano.

[111] Fan X.M., Wang Z.H., Cai T., Yang Y.S., Wu H.C., Cao S., Yang Z.H., Zhang W.X. (2021). An integrated highly stable anode enabled by carbon nanotube-reinforced all-carbon binder for enhanced performance in lithium-ion battery. Carbon.

[112] Fan X.M., Cai T., Wang S.Y., Yang Z.H., Zhang W.X. (2023). Carbon nanotube-reinforced dual carbon stress-buffering for highly stable silicon anode material in lithium-ion battery. Small.

[113] Choi M.H., Sung J.Y., Yeo G.C., Chae S., Ko M. (2023). A strategy of boosting the effect of carbon nanotubes in graphite-blended Si electrodes for high-energy lithium-ion batteries. J. Energy Storage.

[114] Sun B.Y., Wang S., Zhou S.J., Liu J.N., Mao C.W., Liu K.F., Fan H., Xie J.Y., Song J.X. (2024). Biomimetics-inspired architecture enablesthe strength–toughness of ultrahigh-loading silicon electrode. Adv. Funct. Mater..

[115] Ma F., Liu Y.X., Huang T., Du X.R., Lu Q.Q., Kid K. (2024). Facile in situ polymerization synthesis of poly(ionic liquid)-based polymer electrolyte for high-performance solid-state batteries. Energ. Convers. Man-X.

[116] Zhao Y.M., Yue F.S., Li S.C., Li S.C., Zhang Y., Tian Z.R., Xu Q., Xin S., Guo Y.G. (2021). Advances of polymer binders for silicon-based anodes in high energy density lithium-ion batteries. InfoMat.

[117] Yu X.H., Yang H.Y., Meng H.W., Sun Y.L., Zheng J., Ma D.Q., Xu X.H. (2015). Three-dimensional conductive gel network as an effective binder for high-performance Si electrodes in lithium-ion batteries. Acs Appl. Mater. Interfaces.

[118] Zhao H., Du A., Ling M., Battaglia V., Liu G. (2016). Conductive polymer binder for nano-silicon/graphite composite electrode in lithium-ion batteries towards a practical application. Electrochim. Acta.

[119] Salem N., Lavrisa M., Abu-Lebdeh Y. (2016). Ionically-functionalized poly(thiophene) conductive polymers as binders for silicon and graphite anodes for Li-ion batteries. Energy Technol..

[120] Wu H., Yu G.H., Pan L.J., Liu N., McDowell M.T., Bao Z.A., Cui Y. (2013). Stable Li-ion battery anodes by in-situ polymerization of conducting hydrogel to conformally coat silicon nanoparticles. Nat. Commun..

[121] Pan S.Y., Han J.W., Wang Y.Q., Li Z.S., Chen F.Q., Guo Y., Han Z.S., Xiao K.F., Yu Z.C., Yu M.Y. (2022). Integrating SEI into layered conductive polymer coatings for ultrastable silicon anodes. Adv. Mater..

[122] Chen B.Q., Xu D.M., Chai S.M., Chang Z., Pan A.Q. (2024). Enhanced silicon anodes with robust SEI formation enabled by functional conductive binder. Adv. Funct. Mater..

[123] Cai Y.F., Liu C.X., Yu Z.A., Ma W.C., Jin Q., Du R.C., Qian B.Y., Jin X.X., Wu H.M., Zhang Q.H. (2023). Slidable and highly ionic conductive polymer binder for high-performance Si anodes in lithium-ion batteries. Adv. Sci..

[124] Jeong M.G., Ahn S., Yokoshima T., Nara H., Momma T., Osaka T. (2016). New approach for enhancing electrical conductivity of electrodeposited Si-based anode material for Li secondary batteries: Self-incorporation of nano Cu metal in Si–O–C composite. Nano Energy.

[125] Murugesan S., Harris J.T., Korgel B.A., Stevenson K.J. (2012). Copper-coated amorphous silicon particles as an anode material for lithium-ion batteries. Chem. Mater..

[126] Mu T.S., Zhao Y., Zhao C.T., Holmes N.G., Lou S.F., Li J.J., Li W.H., He M.X., Sun Y.P., Du C.Y. (2021). Stable silicon anodes by molecular layer deposited artificial zincone coatings. Adv. Funct. Mater..

[127] Li D., Pan K., Li A.Q., Jiang J.T., Wu Y., Li J.K., Zheng F.H., Xie F.Q., Wang H.Q., Pan Q.C. (2024). Well-dispersed Bi nanoparticles for promoting the lithium storage performance of Si anode: Effect of the bridging Bi nanoparticles. J. Colloid Interf. Sci..

[128] Ipadeola K.A., Abdullah M.A., Eid K. (2024). Recent advances in porous multimetallic alloy-based anodes for rechargeable alkali metal-ion batteries. Energy Mater..

[129] Ding B., Cai Z.F., Ahsan Z., Ma Y.Z., Zhang S.H., Song G.S., Yuan C.Z., Yang W.D., Wen C. (2021). A review of metal silicides for lithium-ion battery anode application. Acta Metall. Sin..

[130] Zhang Q.B., Chen H.X., Luo L.L., Zhao B.T., Luo H., Han X., Wang J.W., Wang C.M., Yang Y., Zhu T. (2018). Harnessing the concurrent reaction dynamics in active Si and Ge to achieve high performance lithium-ion batteries. Energy Environ. Sci..

[131] Liu S., Feng J.K., Bian X.F., Qian Y.T., Liu J., Xu H. (2015). Nanoporous germanium as high-capacity lithium-ion battery anode. Nano Energy.

[132] Yang Y.H., Liu S., Bian X.F., Feng J.K., An Y.L., Yuan C. (2018). Morphology- and porosity-tunable synthesis of 3D nanoporous SiGe alloy as a high-performance lithium-ion battery anode. ACS Nano.

[133] Wei S.Y., Hartman T., Mourdikoudis S., Liu X.T., Wang G., Kovalska E., Wu B., Azadmanjiri J., Yu R.Z., Chacko L. (2024). Reaction mechanism and performance of innovative 2D germanane-silicane alloys: SixGe1-xH electrodes in lithium-ion batteries. Adv. Sci..

